# Impaired benthic macrofauna function 4 years after sediment capping with activated carbon in the Grenland fjords, Norway

**DOI:** 10.1007/s11356-020-11607-0

**Published:** 2020-12-02

**Authors:** Caroline Raymond, Göran S Samuelsson, Stefan Agrenius, Morten T Schaanning, Jonas S Gunnarsson

**Affiliations:** 1 Department of Ecology, Environment and Plant Sciences (DEEP), Stockholm University, 106 91 Stockholm, Sweden; 2 Svensk Ekologikonsult, Skiftesvägen 17, 163 43 Stockholm, Sweden; 3 Department of Marine Sciences–Kristineberg, University of Gothenburg, 451 78 Fiskebäckskil, Sweden; 4 Norwegian Institute for Water Research (NIVA), 0349 Oslo, Norway

**Keywords:** Benthic ecology, Macrofauna, Bioturbation, Bioirrigation, Index, Contaminated sediment, Remediation

## Abstract

The sediments in the Grenland fjords in southern Norway are heavily contaminated by large emissions of dioxins and mercury from historic industrial activities. As a possible in situ remediation option, thin-layer sediment surface capping with powdered activated carbon (AC) mixed with clay was applied at two large test sites (10,000 and 40,000 m^2^) at 30-m and 95-m depths, respectively, in 2009. This paper describes the long-term biological effects of the AC treatment on marine benthic communities up to 4 years after treatment. Our results show that the capping with AC strongly reduced the benthic species diversity, abundance, and biomass by up to 90%. Vital functions in the benthic ecosystem such as particle reworking and bioirrigation of the sediment were also reduced, analyzed by using novel bioturbation and bioirrigation indices (BP_c_, BIP_c_, and IP_c_). Much of the initial effects observed after 1 and 14 months were still present after 49 months, indicating that the effects are long-lasting. These long-lasting negative ecological effects should be carefully considered before decisions are made on sediment remediation with powdered AC, especially in large areas, since important ecosystem functions can be impaired.

**Supplementary Information:**

The online version contains supplementary material available at 10.1007/s11356-020-11607-0.

## Introduction

In coastal marine environments, sediments are often major sinks for contaminants from industrial and municipal activities. Leakage from contaminated sediments may act as new sources to pollution in areas where primary sources have been cleaned or closed down (Larsson 1985). Traditional remediation methods in aquatic environments are dredging or isolation capping. However, dredging is costly and involves moving large amounts of contaminated sediment that need to be deposited elsewhere, and conventional capping use comprehensive isolation layers, e.g., geotextiles or thick layers of sand, in order to prevent the contaminants from being released to the water column (Reible et al. 2008). An alternative capping method proposes a thin cap containing a strong sorbent such as activated carbon (AC) onto the contaminated sediments (Ghosh et al. 2011). AC can sorb the contaminants and reduce their release to the water column and thereby decrease their bioavailability (Cho et al. 2009, 2007; Millward et al. 2005; Patmont et al. 2014; Zimmerman et al. 2004, 2005). One advantage of the remediation method with AC is that lesser amount of capping material is used compared to conventional capping. Another advantage is that the benthic macrofauna could survive a thin cap and facilitate the mixing of the sorbent into the sediment though their reworking activity (bioturbation) and thus increase the capping efficiency (Ghosh et al. 2011; Sun and Ghosh 2007).

In contrast to the positive effects of AC for reducing contaminants’ release and bioavailability, several studies have shown that AC can be harmful to benthic macrofauna (e.g., Janssen and Beckingham 2013; Jonker et al. 2009; Rakowska et al. 2012; Samuelsson et al. 2017). Both AC concentration and particle size seem to be important factors for if, and to what extent, the benthic organisms are affected (Abel and Akkanen 2018, 2019; Kupryianchyk et al. 2013a, 2012; Nybom et al. 2015, 2012). Further, the observed effects of AC seem also to depend on ecosystem conditions such as depth, if it is a limnic or marine system, as well as if it is a laboratory or field study. The reported negative biological effects caused by exposure to AC are sometimes severe, for example reduced survival (Kupryianchyk et al. 2013a, 2011; McLeod et al. 2008), inhibited growth (Janssen et al. 2012; Kupryianchyk et al. 2011; McLeod et al. 2008; Millward et al. 2005; Nybom et al. 2015, 2012), behavioral changes (Jonker et al. 2009; Nybom et al. 2015, 2012), reproduction interferences (Nybom et al. 2015, 2012), and morphological changes (Nybom et al. 2015).

Such negative biological consequences are important from an ecological point of view as benthic fauna substantially influence the sediment with their activities, e.g., ingestion, defecation, irrigation, and burrowing. These activities are examples of bioturbation, i.e., the process of organisms’ particle mixing by reworking and exchange of solute and water in burrows (Kristensen et al. 2012). Bioturbation is a vital process in the soft bottom ecosystems, influencing for example geochemical gradients, microbial community structures, and redistribution of food resources within the sediment, as well as regulating the rate of gas exchange and nutrient fluxes with the overlying water (Aller 1994; Meysman et al. 2006; Rhoads 1974). Because different species affect the sediment properties in different ways, the functioning of the sediment ecosystem is dependent on the structure of the benthic community (Dauwe et al. 1998; Gray 1974; Pearson and Rosenberg 1978). From an ecosystem perspective, it is therefore of great importance to find out if a thin layer of AC would have any long-term harmful effects on the benthic fauna, and to quantify potential functional changes in terms of bioturbation activities.

In this study, we assessed the long-term effects of thin-layer capping with powdered AC on marine benthic macrofauna communities 49 months (4 years) after capping. The results follow up the initial effects of AC capping on benthic macrofauna that are presented in Samuelsson et al. (2017), which reported negative effects after 1 and 14 months on number of species, total abundance, and biomass. The study was conducted as a large-scale field experiment in the Grenland fjords in southeast Norway, where the sediment is heavily contaminated by dioxins, furans, and mercury from past industrial activities. One unique and novel aspect in this paper is the long-term perspective, where the effects of AC capping on the benthic community are evaluated in situ 4 years after capping. Another novelty is that we assessed the changes on the benthic communities using three recently developed bioturbation indices. In the first index, the potential reworking process is quantified by calculating the community bioturbation potential, BP_c_ (Solan et al. 2004). In the second and third indices, the potential exchange of solutes at the sediment-water interface is quantified by calculating the community bioirrigation potentials, BIP_c_ (Renz et al. 2018) and IP_c_ (Wrede et al. 2018). The use of these indices gives an estimate of how AC induces changes on the benthic community structure result in degradation of essential ecosystem functions such as nutrient cycling, mineralization of organic matter and oxygenation of the sediment.

## Materials and methods

### Study area and study design

**Fig. 1:**
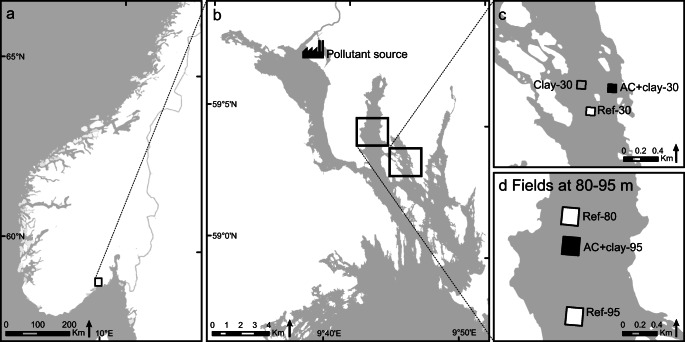
Map of the study area. (a) The Grenland fjords are located in southeast Norway. (b) The main pollutant source, the magnesium smelter, was situated in the inner part of the fjord system. (c) The experimental fields at 30-m are located in the Ormerfjord, and (d) the experimental fields at 80–95-m depth are located in the Eidangerfjord

The Grenland fjords are situated in southeast Norway (59°02′N, 009°43′E). The sediments in the fjords are heavily polluted from industrial activities situated in the innermost part of the fjord area (Fig. 1). The discharges of wastewater from a magnesium factory operating 1951–2002 have contaminated the sediments in the fjord area with mercury (Hg) and persistent organic pollutants (POPs) such as polychlorinated dibenzofurans/dibenzo-p-dioxins (PCDFs/PCDDs), hexachlorobenzene (HCB), and octrachlorostyrene (OCS), and other industrial activities in the area have in addition contaminated the sediments with polychlorinated biphenyls (PCBs) (Knutzen et al. 2003). The high contamination load in the fjord has led to several restrictions on seafood consumption in the area and to suggestions of various remediation scenarios (Ruus et al. 2006; Saloranta et al. 2008). Since the fjord area is too large to be dredged or capped using conventional capping, thin-layer capping with AC was proposed as an alternative remediation technique. In 2009, one of the largest thin-layer capping experiments in the world was launched in two outer arms of the Grenland fjords, Norway.

At 30-m depth in the Ormerfjord, two test fields (100 × 100 m, i.e., 10,000 m^2^) were capped with either AC mixed with clay (AC + clay-30) or only clay (Clay-30). A third field at 30-m depth was defined in the area and serves as an uncapped reference field (Ref-30). The field capped with only clay also serves as a reference in order to distinguish the effects of the added AC from the effects of capping with only clay, and no initial negative effects of capping with only clay were observed on the benthic fauna (Samuelsson et al. 2017). At 95-m depth in the Eidangerfjord, a larger test field (200 × 200 m, i.e., 40,000 m^2^) was capped with AC mixed with clay (AC + clay-95). An uncapped reference field was defined at 80-m depth (Ref-80). The location of AC + clay-95 and Ref-80 was chosen to avoid any risk of disturbance from trawling activities. As trawling ceased in the fjord after establishment of the field experiment, another uncapped reference field at 95-m depth (Ref-95) was introduced after 14 months to confirm that effects were due to treatment rather than to the different depths of 80 and 95-m. A general description of the fjord environment and dominating benthic species are presented in Samuelsson et al. (2017).

### Capping material

The clay material used in the capping material in Clay-30 and both AC + clay fields was suction dredged from 10-m depth in the inner part of the Ormerfjord. The top 10 cm were discarded to acquire a cleaner sediment (< 1 ng kg^−1^ TEQ) and to eliminate benthic fauna (Cornelissen et al. 2012). For the AC + clay treatment, powdered AC was used (Jacobi Carbons, BP2 fine powder; average particle size of 20 μm, 80% smaller than 45 μm). To obtain the AC + clay slurry, AC and clay were mixed in a 275 m^3^ tank on a 42-m-long trailing suction hopper dredger (Cornelissen et al. 2012). The target AC concentration was 10% of dry weight (d.w.) clay, which was achieved in the slurry used at 30-m depth, whereas the AC + clay slurry used at 95-m depth only reached an AC concentration of 7% of d.w. clay. The capping slurries were pumped out from the tank using a long hose held 5–10-m above the sediment surface. The AC particles did not settle as expected and up to 75% were lost to surrounding areas due to lateral advection (Cornelissen et al. 2012). The thickness of the caps were after 1 month measured to 11 ± 6 mm in AC + clay-30, 12 ± 3 mm in AC + clay-95, and 37 ± 11 mm in Clay-30 (Eek et al. 2011). A more detailed description of how the capping materials were applied and their effects on reducing contaminant fluxes is presented in Cornelissen et al. (2012, 2015).

### Sediment analyses

Three sediment Kajak cores (inner diameter 80 mm) from each field were sampled 49 months after capping and sliced into a surface (0–2 cm) and a subsurface (2–5 cm) section for ulterior sediment analyses. Total carbon content (TC), total organic carbon (TOC), and total nitrogen (TN) were analyzed using an elemental analyzer (Thermo Scientific Flash 2000) in our lab at Stockholm University. For the TOC samples, the sediment were pre-treated with hydrochloric acid (HCl) to eliminate carbonates according to Hedges and Stern (1984). AC content in the two AC fields was calculated by subtracting the mean TOC and TC contents from the respective reference fields (Ref-30 and Ref-95). A TOC/AC ratio was calculated to estimate relative differences of a hypothesized sequestration of natural available sediment organic carbon by the added AC. This potential side effect has previously been suggested from the perspective of AC performance (Amstaetter et al. 2012; Kwon and Pignatello 2005), and from the potential negative effect to benthic fauna (Jonker et al. 2004; Samuelsson et al. 2017).

### Sampling and analyses of the benthic fauna

The fields were capped in September 2009, and benthic macrofauna were sampled after 1 month (13–14 October 2009), 14 months (8–9 November 2010), and 49 months (23–24 October 2013). In the first sampling event, 3 replicate van Veen grabs (0.1 m^2^) were taken in each field. To improve the statistical power, 5 replicate grabs per field were taken at the second and third sampling events. All the grab samples had full volume (19 l), were immediately washed through a 1-mm sieve and the retained material was preserved in 4% buffered formaldehyde. The benthic macrofauna was identified to species level, or in few exceptions, to the lowest possible taxonomic level. In each sample, abundance (number of individuals) and biomass (g wet weight) were determined per taxon; see supplementary material Table S-1 for a complete list of all taxa. The field sampling and subsequent sample processing were conducted according to the guidelines of the European standard ISO 16665: 2014.

### Bioturbation and bioirrigation indices

The bioturbation and bioirrigation indices were calculated from the species’ potential for particle reworking and solute exchange. Bioturbation as definition often includes both particle reworking and the associated bioirrigation (Kristensen et al. 2012), but are in these indices separated as different processes. The potential reworking activity is estimated with the community bioturbation potential index, BP_c_ (Solan et al. 2004; Swift 1993). For simplicity, we have used the term bioturbation for particle reworking referring to results of the bioturbation index. BP_c_ is based on species’ mean individual biomass, abundance, and categorical classifications of species mobility (*M*_i_) and reworking (*R*_i_). Most of the *M*_i_ and *R*_i_ scores we used are from Queirós et al. (2013). However, out of our 182 taxa, 53 taxa had no previous *M*_i_ and *R*_i_ classifications. Of these 53 taxa, we found the values from closely related species or of the family suitable for 33 taxa, and the remaining 20 taxa were classified according to literature or in some cases our own knowledge. Further, we reclassified 34 *M*_i_ values and 41 *R*_i_ values from Queirós et al. (2013), also based on literature or our own knowledge. This reclassification had only marginal effects on the overall result (see supplementary material Fig. S-1 for BP_c_ based on Queirós original values). A complete list of species with original and new classifications is provided in the supplementary material (Table S-2). The definitions for *R*_i_ and *M*_i_ trait values are presented in Table 1.

**Table 1:** The bioturbation index BP_c_ categorical values of traits in reworking (*R*_i_), and mobility (*M*_i_). Definitions from Queirós et al. (2013)

Trait	Score	BP_c_ mode
Reworking	1	Epifauna that bioturbate at the sediment-water interface
	2	Surficial modifiers (activities restricted to upper 2 cm)
	3	Upward and downward conveyors
	4	Biodiffusers
	5	Regenerators that excavate holes, transferring sediment at depth to the surface
Mobility	1	Lives in fixed tubes
	2	Limited movement
	3	Slow, free movement through the sediment matrix
	4	Free three-dimensional movement via burrow system

The community bioturbating potential index BP_c_ is defined as:

$$ {\mathrm{BP}}_{\mathrm{c}}=\sum \limits_{i=1}^n\sqrt{B_i/{A}_i}\times {A}_i\times {M}_i\times {R}_i $$

where *B*_i_ is the biomass (wet weight) and *A*_i_ is the abundance of taxon i in a sample, and *M*_i_ and *R*_i_ are categorical classifications of species’ mobility and reworking, respectively (Solan et al. 2004).

The potential irrigation activity was calculated using two indices: the community bioirrigation potential, BIP_c_ (Renz et al. 2018), and the irrigation potential, IP_c_ (Wrede et al. 2018). Both indices are based on species mean individual biomass, abundance, and categorical classifications of species feeding type, burrow type, and burrow depth. However, the categorical classifications are different in the two indices (Table 2). Further, there is a difference in the scaling of individual biomass between the two indices. The two indices are, however, both based on species traits for diffusion and advection in the sediment. In contrast to the bioturbation index BP_c_, the two bioirrigation indices are based on biomass expressed as ash-free dry weight (AFDW). To obtain AFDW, our wet weight values were recalculated using conversion factors from the literature (Brey 2001; Brey et al. 2010; Ricciardi and Bourget 1998). The recalculation of wet weight to AFDW is proposed by both Renz et al. (2018) and Wrede et al. (2018) if the biomass is not expressed as AFDW originally. See supplementary material Table S-2 for AFDW conversion factors as well as the species classifications for the bioirrigation indices.

**Table 2:** The bioirrigation indices BIP_c_ and IP_c_ trait categories, their modes, and scores. BIP_c_ definitions from Renz et al. (2018) and IP_c_ definitions from Wrede et al. (2018)

Trait	Score	BIPc mode	Score	IPc mode
Feeding	1	Predator, scavenger, herbivore, omnivore	1	Surface filter feeder
	2	Deposit feeder, facultative deposit/suspension feeder	2	Predator
	3	Suspension feeder siphon	3	Deposit feeder
	4	Suspension feeder without siphon	4	Subsurface filter feeder
	5	Subsurface deposit feeder		
Burrowing	0	Attached, epifauna	0	No activity*
	1	Free living	1	Epifauna, internal irrigation (e.g., siphons)
	2	Living in a fixed tube	2	Open irrigation (e.g., U- or Y-shaped burrows
	3	Living in a burrow	3	Blind ended irrigation (e.g., blind ended burrows, no burrow system)
Depth	0	Epifauna	0	Epifauna*
	1	0–1 cm*	1	0–2 cm
	3	1–3 cm*	2	2–5 cm
	5	3–5 cm*	3	5–10 cm
	7	5–7 cm*	4	> 10 cm
	10	7–10 cm*		
	15	10–15 cm*		
	20	15–20 cm* (or deeper)		

*Modified or new term, not existing in the original index

The bioirrigation index BIP_c_ is defined as:

$$ {\mathrm{BIP}}_{\mathrm{c}}=\sum \limits_{i=1}^n\sqrt{B_i/{A}_i}\times {A}_i\times {FT}_i\times {BT}_i\times {L}_i $$

where *B*_i_ is the biomass (AFDW) and *A*_i_ is the abundance of taxon *i* in a sample, FT_i_ is the feeding type, BT_i_ is the burrow type, and *L*_i_ is the length of the species burrow (Renz et al. 2018). For the *L*_i_, we used the depth where the species often live in the sediment instead of the maximum depth recorded in the literature as proposed by Renz et al. (2018).

The alternative irrigation index IP_c_ is defined as:

$$ {\mathrm{IP}}_{\mathrm{c}}=\sum \limits_{i=1}^n{\left(\frac{Bi}{Ai}\right)}^{0.75}\times {A}_i\times {FT}_i\times {BT}_i\times {ID}_i $$

where *B*_i_ is the biomass (AFDW) and *A*_i_ is the abundance of taxon *i* in a sample, FT_i_ is the feeding type, BT_i_ is the burrow type, and ID_i_ is the depth of the species burrow (Wrede et al. 2018).

The weighting of the individual biomass is different in the IP_c_ index compared to the other two indices. Biomass and abundance are set to contribute equally in the BP_c_ and BIP_c_ indices (since $\sqrt{B_i/{A}_i}\times {A}_i$ = ${B}_i^{\kern0.5em 0.5}\times {A}_i^{\kern0.5em 0.5}$), whereas in the IP_c_ index the biomass (B_*i*_^0.75^) is weighted considerably higher than the abundance (${A}_i^{\kern0.5em 0.25}$, since ${A}_i^{-0.75}\times {A}_i={A}_i^{\kern0.5em 0.25}$).

### Statistical analyses

The data from the shallow (30-m) fjord and the deeper (80–95-m) fjord were treated separately in all statistical analyses. Besides the difference in depth, the fjords are located in two different areas in the Grenland fjords, which differ in hydrography, sediment, and benthic community type. To test the effects of treatments and time, permutational analysis of variance (PERMANOVA; Anderson 2001) was used on univariate metrics, i.e., abundance, biomass, and number of species, as well as for the bioturbation and bioirrigation indices BP_c_, BIP_c_, and IP_c_, using the statistical software package PRIMER 6 with the PERMANOVA+ add-on (Plymouth Laboratories, England). Euclidian distance was used and with suitable transformations to achieve homogenous variances. Pairwise post hoc tests were carried out with planned comparisons using the same PERMANOVA procedures (equivalent to Dunnett’s post hoc test in a traditional ANOVA), and Monte-Carlo sampling was used if the numbers of unique permutations were low. The significance level for all statistical tests was set at *α* = 0.05. The focus of this article has been to scrutinize the effects from AC at all sampling occasions rather than the overall results in the PERMANOVA main test, whereby we present the pairwise tests in the article and refer to the supplementary material Table S-3 for the results of the overall main test.

Correlation tests were performed on the biological metrics and indices using distance based linear models (DistLM in PERMANOVA+ for PRIMER 6). The purpose was mainly to investigate how the outcome (*R*^2^ value) of the indices were related to each other and to the input variables abundance and biomass, as well as to the indirect numbers of species variable, in the examples in this study. Therefore, their *R*^2^ values were of interest rather than their significance. Data were transformed and checked for linearity, homoscedasticity, and normality in distribution.

## Results

### Sediment data

Surface sediment data 49 months after capping are presented in Table 3. The total carbon (TC) and total organic carbon (TOC) contents in the reference fields were higher in the 80–95-m area (2.5–3.4%) than in the 30-m area (1.4–1.8%). Further, the TC, TOC, and C/N ratios were higher in the AC fields compared to the reference fields, showing that AC still accounted for substantial part of the carbon content in the surface sediment in these fields even 49 months after capping. The average AC content in AC + clay-30 was 1.9% in the upper 0–2 cm and 2.0% in the 2–5-cm layer, and in AC + clay-95 the average AC content was 0.8% in the upper 0–2 cm and 1.3% in the 2–5-cm layer. This shows that AC has been mixed into the deeper layers in the sediment. The calculated TOC/AC ratio presents a large difference between the two depths, where the AC-capped field at 30-m only had approximately one fourth of the calculated TOC/AC compared to the AC field at 95-m depth.

**Table 3:** Sediment data 49 months after capping. Total carbon (TC), total organic carbon (TOC), total nitrogen (TN), and the C/N ratio (ratio of TC and TN) are on average 0–5 cm since there were no significant differences between the upper and lower fractions. The TC and TOC values in the AC fields include the AC content, explaining the high values. The AC content (%) and TOC/AC ratio are shown in the intervals of 0–2 cm and 2–5 cm. The TOC/AC ratios are calculated using the TOC value from Ref-30 and the average of Ref-80 and Ref-95

Depth	Field	TC (%)	TOC (%)	TN (%)	C/N	AC (%)	TOC/AC ratio
30 m	Ref-30	1.5 ± 0.1	1.4 ± 0.11	0.13 ± 0.01	11.5	– | –	– | –
	Clay-30	1.8 ± 0.3	1.5 ± 0.34	0.14 ± 0.03	12.7	– | –	– | –
	AC + clay-30	3.5 ± 0.4	3.3 ± 0.37	0.19 ± 0.05	19.4	1.9 | 2.0	0.7 | 0.7
80–95 m	Ref-80	3.0 ± 0.1	2.5 ± 0.12	0.22 ± 0.02	13.3	– | –	– | –
	Ref-95	3.4 ± 0.1	2.7 ± 0.10	0.24 ± 0.02	14.6	– | –	– | –
	AC + clay-95	4.2 ± 0.3	3.7 ± 0.36	0.24 ± 0.02	17.8	0.8 | 1.3	3.3 | 2.0

### Number of species, abundance, and biomass

In total, 182 taxa were identified from 7990 specimens found in the 75 grab samples. At 30-m, 92 taxa were identified from 2497 specimens in the 39 grab samples. At 80–95-m, 149 taxa were identified from 5493 specimens in the 36 grab samples.

**Fig. 2:**
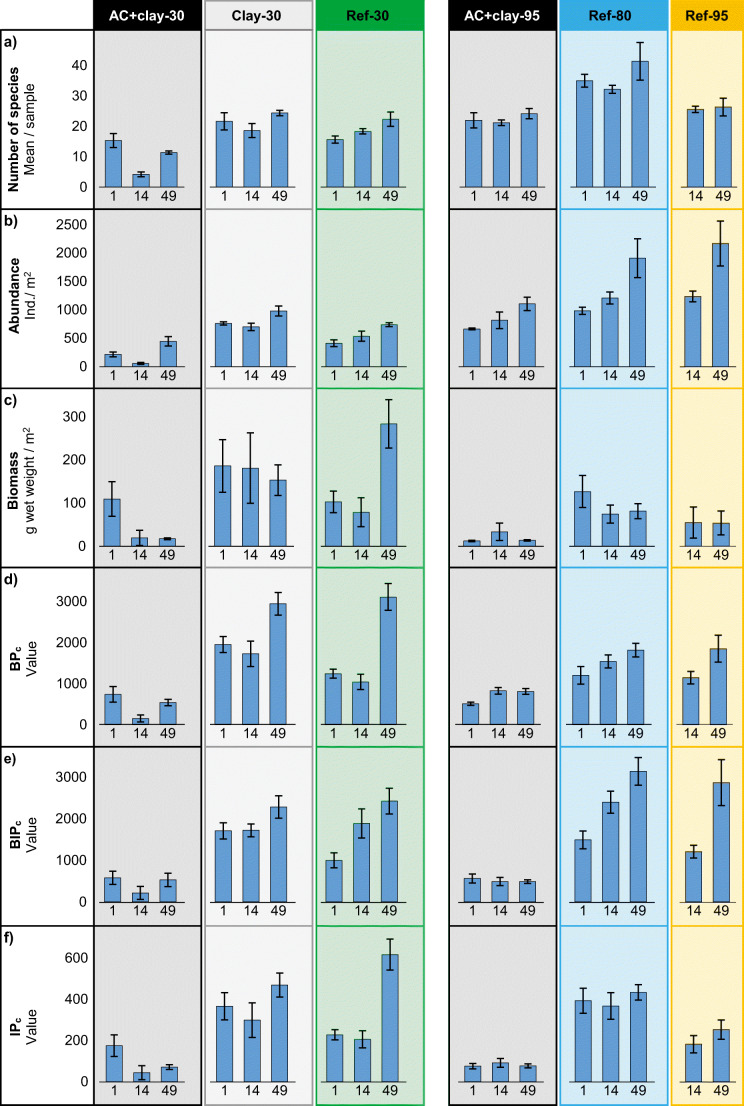
Biometrics 1, 14, and 49 months after capping. (a) Number of species per sample, (b) organism abundance per square meter, (c) biomass (g wet weight) per square meter, (d) bioturbation index BP_c_, (e) bioirrigation index BIP_c_, and (f) bioirrigation index IP_c_

In AC + clay-30, the number of species, abundance, and biomass was significantly lower compared to Ref-30 and Clay-30 (Fig. 2, Table S-3). In the following post hoc tests, only the abundance was significantly lower after 1 month, but after 14 and 49 months, the number of species and biomass was also significantly lower in AC + clay-30 than in Ref-30 and Clay-30 (Table 4). After 14 months, all response variables in AC + clay-30 showed very low values, only 8–23% compared to Ref-30 and Clay-30 (Table 5). After 49 months, the number of species and abundance increased in AC + clay-30, but remained significantly lower compared to Ref-30 and Clay-30. The biomass in AC + clay-30 remained low after 49 months, with only 6–11% of the biomass in Ref-30 and Clay-30. The reference and capping control field, Ref-30 and Clay-30, were not significantly different regarding the number of species and biomass; however, Clay-30 had significantly higher abundance than Ref-30 after 1 and 49 months (Fig. 2, Table 4).

**Table 4:** Results from the PERMANOVA post hoc test between treatments. Significant *p* values are shown in bold numbers, *α* = 0.05. *PsF* pseudo F value, *P(MC) p* values by Monte-Carlo sampling. For the PERMANOVA overall main test, see supplementary material Table S-3

		No. of species		Abundance		Biomass		BP_c_		BIP_c_		IP_c_	
Fields	Month	*PsF*	P(MC)	*PsF*	P(MC)	*PsF*	P(MC)	*PsF*	P(MC)	*PsF*	P(MC)	*PsF*	P(MC)
AC + clay-30 vs Ref-30	1	*0.02*	0.9051	*7.75*	**0.0479**	*0.00*	0.9557	*4.69*	0.0991	*2.88*	0.1664	*0.91*	0.3979
	14	*144.03*	**0.0001**	*51.69*	**0.0002**	*5.34*	**0.0473**	*24.14*	**0.0016**	*25.33*	**0.0012**	*11.10*	**0.0112**
	49	*20.79*	**0.0018**	*10.13*	**0.0134**	*57.39*	**0.0003**	*82.68*	**0.0001**	*31.53*	**0.0008**	*80.64*	**0.0001**
AC + clay-30 vs Clay-30	1	*2.96*	0.1659	*73.60*	**0.0008**	*0.78*	0.4201	*17.08*	**0.0137**	*16.58*	**0.0137**	*4.86*	0.0958
	14	*35.15*	**0.0002**	*144.89*	**0.0001**	*11.11*	**0.0108**	*39.11*	**0.0003**	*37.23*	**0.0002**	*13.33*	**0.0060**
	49	*165.69*	**0.0001**	*19.48*	**0.0023**	*46.15*	**0.0003**	*102.91*	**0.0001**	*36.23*	**0.0002**	*78.42*	**0.0001**
Clay-30 vs Ref-30	1	*3.77*	0.126	*22.44*	**0.0090**	*1.04*	0.3641	*11.36*	**0.0303**	*6.95*	0.0595	*4.10*	0.1186
	14	*0.01*	0.9373	*2.35*	0.1657	*1.46*	0.2514	*3.46*	0.1035	*0.08*	0.7825	*0.88*	0.3868
	49	*0.63*	0.452	*7.02*	**0.0303**	*3.11*	0.1136	*0.12*	0.7453	*0.08*	0.7912	*2.12*	0.1793
AC + clay-95 vs Ref-80	1	*12.28*	**0.0269**	*32.48*	**0.0042**	*53.57*	**0.0015**	*20.89*	**0.0123**	*17.05*	**0.0148**	*55.10*	**0.0018**
	14	*47.75*	**0.0003**	*4.37*	0.0682	*5.34*	**0.0461**	*15.89*	**0.0039**	*34.56*	**0.0006**	*24.66*	**0.0014**
	49	*8.30*	**0.0202**	*5.22*	**0.0493**	*52.32*	**0.0002**	*39.83*	**0.0004**	*161.45*	**0.0002**	*124.57*	**0.0001**
AC + clay-95 vs Ref-95	14	*10.20*	**0.0136**	*4.93*	0.0587	*0.34*	0.5787	*3.23*	0.1078	*11.08*	**0.0110**	*6.33*	**0.0333**
	49	*0.34*	0.5715	*5.38*	**0.0457**	*9.04*	**0.0154**	*12.72*	**0.0074**	*51.51*	**0.0001**	*25.55*	**0.0014**
Ref-80 vs Ref-95	14	*15.83*	**0.0037**	*0.04*	0.8605	*2.51*	0.1564	*3.12*	0.1165	*14.89*	**0.0069**	*6.23*	**0.0372**
	49	*4.59*	0.0641	*0.13*	0.7365	*3.57*	0.1000	*0.03*	0.8732	*0.39*	0.5359	*7.93*	**0.0213**

**Table 5:** Difference of AC + clay values expressed in percentage of the values in the reference fields. Significant differences from Table 4 are marked with an asterisk (*)

	Month	AC + clay-30 vs Ref-30	AC + clay-30 vs Clay-30	AC + clay-95 vs Ref-80	AC + clay-95 vs Ref-95
No. of species	1	98%	71%	63%*	–
	14	23%*	23%*	66%*	83%*
	49	51%*	47%*	58%*	92%
Abundance	1	52%*	28%*	67%*	–
	14	10%*	8%*	68%	66%
	49	60%*	45%*	58%*	51%*
Biomass	1	107%	59%	9%*	–
	14	24%*	11%*	45%*	61%
	49	6%*	11%*	17%*	25%*
Bioturbation BP_c_	1	59%	38%*	42%*	–
	14	14%*	9%*	54%*	72%
	49	17%*	18%*	44%*	44%*
Bioirrigation BIP_c_	1	58%	34%*	38%*	–
	14	11%*	12%*	20%*	40%*
	49	22%*	23%*	15%*	17%*
Bioirrigation IP_c_	1	77%	48%	19%*	–
	14	21%*	15%*	25%*	50%*
	49	11%*	15%*	18%*	30%*

In AC + clay-95, the number of species, abundance, and biomass was significantly lower compared to Ref-80 (Fig. 2, Table S-3). Compared to Ref-95, AC + clay-95 had lower abundance and biomass (Fig. 2, Table S-3). The post hoc tests shows that the abundance and biomass in AC + clay-95 was significantly lower than in Ref-95 after 49 months, but not after 14 months (Table 4). The biomass in AC + clay-95 was remarkably low already after 1 month, only 9% of the biomass in Ref-80 (Table 5). After 14 months, the biomass in AC + clay-95 increased, reaching 45–61% of the biomass in the reference fields. However, the biomass in AC + clay-95 decreased after 49 months, resulting in a biomass between 17 and 25% of the reference values. The abundance increased with time in all fields at 80–95-m, but was consistently lower in AC + clay-95 (51–68%) compared to the reference fields (Fig. 2, Table 5). The two reference fields, Ref-80 and Ref-95, were only significantly different in the number of species after 14 months; otherwise, the number of species as well as the abundance and biomass was not significantly different between the two fields (Table 4). Ref-80 had overall higher number of species than Ref-95 (Fig. 2).

### Bioturbation and bioirrigation indices

The values of the bioturbation index BP_c_ and the bioirrigation indices BIP_c_ and IP_c_ in AC + clay-30 were all significantly lower than those in Ref-30 and Clay-30 (Fig. 2, Table S-3). The post hoc tests showed difference only to Clay-30 after 1 month, but after 14 and 49 months, the sediment reworking values in AC + clay-30 were lower compared both to Clay-30 and Ref-30 (Table 4). The sediment reworking activities in AC + clay-30 were found to be very low after 14 months, with only a small increase after 49 months. Compared to Ref-30 and Clay-30, the AC + clay-30 field had between 9 and 18% bioturbation and 11–23% bioirrigation activities after 14 and 49 months (Table 5). Further, the BP_c_ value in AC + clay-30 was after 49 months up to 75% associated with species with protective shells or tubes, compared to 6% in Ref-30 and Clay-30. The Ref-30 and Clay-30 fields showed no overall significant difference in any of the indices (Table S-3); however, in the post hoc tests, the bioturbation index BP_c_ was significantly higher in Clay-30 compared to Ref-30 after 1 month (Table 4).

In AC + clay-95, the bioturbation index BP_c_ and bioirrigation indices BIP_c_ and IP_c_ were overall significantly lower than those in both Ref-80 and Ref-95 (Fig. 2, Table S-3). The post hoc tests revealed significant lower bioturbation and bioirrigation values in AC + clay-95, except for BP_c_ compared to Ref-95 after 14 months (Table 4). The bioturbation activities in AC + clay-95 were measured to around half of the values in the reference fields (Fig. 2, Table 5). The bioirrigation activities in AC + clay-95 were even lower, especially after 49 months when the irrigation was measured to 15–30% of the reference fields (Table 5). The reference fields, Ref-80 and Ref-95, were not significantly different in their bioturbation index BP_c_ (Table 4). In the irrigation indices BIP_c_ and IP_c_, Ref-80 and Ref-95 were significantly different after 14 months, and for IP_c_ also after 49 months (Table 4).

Correlation tests were performed on the indices in order to determine the contribution of abundance and biomass (ww or AFDW) as well as the indices correlation to each other (Table 6). The IP_c_ index had very high correlations to AFDW at both depths (*R*^2^ 0.93–0.94), reflecting the upscaled contribution from the biomass in this index. The BP_c_ and BIP_c_ indices had instead a more similar contribution from abundance and biomass. The indices were rather correlated to each other (*R*^2^ 0.71–0.94). Further, the recalculated AFDW was highly correlated to wet weight biomass, showing the close relationship between these variables.

**Table 6:**
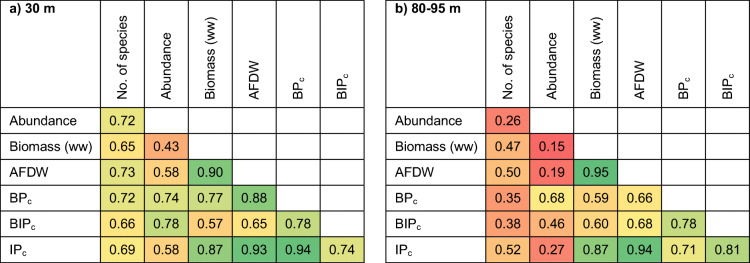
Correlations of the input variables abundance and biomass to the respective indices, and indices’ correlations to each other in (a) 30-m depth and (b) 80–95-m depth. Green indicate high correlation, red indicate low correlation. Note that BPc use wet weight (ww) biomass, while BIPc and IPc use ash-free dry weight (AFDW)

### Community structure

**Fig. 3:**
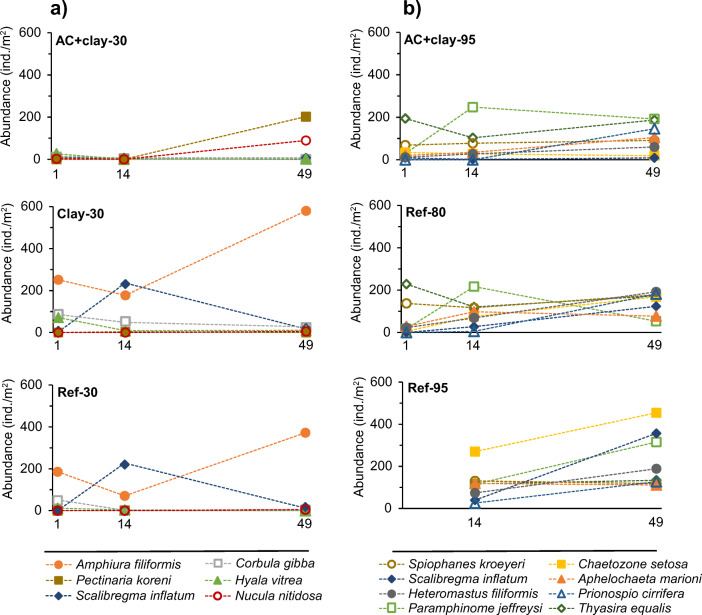
Abundance per square meter for species with the most prominent changes between AC and reference fields 1, 14, and 49 months after capping at (a) 30-m depth and (b) 80–95-m depth

Ref-30 and Clay-30 had in general similar community structures (Fig. 3 and supplementary material Table S-4). The abundance in these two fields was dominated by the brittle star *Amphiura filiformis*, especially after 49 months, when this species exceeded 50% of the total abundance. After 14 months, both fields also had high abundance of the polychaete *Scalibregma inflatum*. AC + clay-30 had, on the other hand, a very different community structure compared to Ref-30 and Clay-30. Both *A. filiformis* and *S. inflatum* disappeared following the capping with AC. After 14 months, the depleted community had very low abundances of all species. However, after 49 months, the AC + clay-30 field had been colonized by the polychaete *Pectinaria koreni* (202 ind/m^2^) and the bivalve *Nucula nitidosa* (89 ind/m^2^), which together accounted for 66% of the total abundance. The biomass (ww) in both Ref-30 and Clay-30 was predominantly made up of sea urchins (*Brissopsis lyrifera*, *Echinocardium cordatum*, and *E. flavescens*) because of these species’ large size. In total, the sea urchins constituted up to 80% of the total biomass in Ref-30 and Clay-30. The biomass in AC + clay-30 was not initially impacted by AC; both *B. lyrifera* and *E. cordatum* were found after 1 month, which sustained the biomass at this time. However, after 14 months, only one specimen of *B. lyrifera* was found, of a relatively small size. After 49 months, no sea urchins at all were found in the AC + clay-30 field.

The most abundant species in Ref-80 and Ref-95 were the mollusk *Thyasira equalis*, together with the polychaetes *Spiophanes kroeyeri*, *Heteromastus filiformis*, and *Aphelochaeta marioni* (Fig. 3 and supplementary material Table S-4). The increased abundance with time was mainly due to higher abundance of polychaetes, e.g., *Paramphinome jeffreysi* (only at 14 months), *Chaetozone setosa* (both at 14 and 49 months), *S. inflatum*, and *Prionospio cirrifera* (only at 49 months). The deeper capped field, AC + clay-95, had in general the same most abundant species as the reference fields, but with lower densities. The AC + clay-95 field had for example lower abundance of the deposit-feeding polychaetes *S. kroeyeri*, *H. filiformis*, *S. inflatum*, and *Ch. setosa* compared to the reference fields. However, the AC + clay-95 field had also a similar strong abundance increase of *P. jeffreysi* after 14 and 49 months, as well as *P. cirrifera* and *A. marioni* after 49 months. *T. equalis* seemed to be unaffected by the capping with AC + clay and had similar abundances as the reference fields on all three sampling occasions, possibly due to their nutritional symbioses with chemosynthetic bacteria. The biomass in the deeper fjord reference fields was also dominated by the sea urchins but they were fewer and larger compared to in the shallower fjord. However, the second and third most important contributors to the biomass in Ref-80 were the relatively large polychaetes *Lipobranchius jeffreysii* and *Streblosoma bairdi*, while these species occurred in lower quantities in Ref-95. Instead, the second most important contributors to the biomass in Ref-95 were the relatively large but scarce polychaete *Glycera unicornis* after 14 months, and the abundant polychaete *S. inflatum* after 49 months. In AC + clay-95, the only finding of sea urchins were after 14 months, with one specimen of *B. lyrifera*. This finding contributed to the higher biomass after 14 months compared to after 1 and 49 months. Otherwise, the most important contributors to the biomass in AC + clay-95 were the abundant mussel *T. equalis* and the polychaete *S. kroeyeri*, in combination with species that fluctuated over time, e.g., the abundant mollusks *Yoldiella. philippiana* after 1 month, *A. nitida* after 14 months, and the relatively abundant polychaete *A. marioni* together with the scarce but relatively large *G. unicornis* after 49 months. The overall community structure in AC + clay-95 was more closely related to the reference field at 95-m that at 80-m depth.

## Discussion

The initial negative response of AC on benthic macrofauna described in Samuelsson et al. (2017) was in this study proven to be long-lasting. At 30-m depth, the observed negative effects were most severe after 14 months, followed by an initial recolonization after 49 months. However, the sediment reworking activity of the benthic communities expressed as potential bioturbation and bioirrigation was still considerably reduced after 49 months, showing that essential ecosystem functions, e.g., nutrient cycling, may still have been altered. The effects in the deeper AC + clay field was not as severe after 14 months as in the shallower AC + clay field, but while there were some signs of improvements in the shallower AC + clay-30, no indication of recovery was observed in the deeper AC + clay-95, making the long-lasting negative effects up to 49 months almost as severe in the deeper site as in the shallower.

### Number of species, abundance, and biomass

In the fjord at 30-m depth, only 10% of the abundance, biomass, and number of species were observed after 14 months in the AC field compared to the reference and clay fields. After 49 months, a small increase was observed in the number of species as well as the abundance, which imply a start of a recolonization process. However, the biomass was still low, indicating that the recolonization was only at the beginning, with the absence of larger and long-lived species (Pearson and Rosenberg 1978). For example, the brittle star *A. filiformis* disappeared initially from AC + clay-30 and were still not found after 49 months, whereas they accounted for up to 50% of the total abundance in Ref-30 and Clay-30. The regained abundance in AC + clay-30 after 49 months was mainly due to colonization of the tube-living polychaete *P. koreni* and the bivalve *N. nitidosa*, which together accounted for more than half of the total abundance. The specimens of *P. koreni* were small and probably settled some months earlier during the spring or summer, while the specimens of *N. nitidosa* most probably had settled 1 year before. *P. koreni* is short lived, reaches maturity within 1 year, and is capable of rapid recolonization through larval recruitment, often resulting in a population maximum during autumn/winter (Arntz and Rumohr 1986). *N. nitidosa* has a longer life span (up to 10 years), reaches maturity after 2–3 years, and produces lecithotrophic larvae with short (2–10 days) planktonic stages which can result in strong recruitments from nearby populations when situations are suitable (Sabatini and Ballerstedt 2008). The traits of these two species makes them among the species that often are found in elevated densities in transitional communities (Pearson and Rosenberg 1978). Common for these two species, and for several of the few other species still present in the AC field, was the presence of protected shells or tubes, which may give a shield from dermal exposure to fine particles of AC (see Fig. S-1c and Table S-2).

In the deeper fjord, AC also negatively affected the number of species, abundance, and biomass. The reductions were significant in all biometrics already after 1 month; however, in contrast to 30-m, the reductions after 14 months were not as drastic and the AC + clay-95 field had in general the same most abundant species as the reference fields. Nonetheless, after 49 months, the total abundance in AC + clay-95 only reached about half of the reference fields’ values, and the biomass reached only a fifth, indicating a poorer community in the AC field. This could be a result of lower recruitment, e.g., less favorable conditions for new larvae to settle in the presence of AC. It could also be that some species have difficulties in coping with the AC and therefore either move away from the field or die off if they find AC capping disturbing or even harmful. Species with protective shells or tubes were more abundant in the deeper fjord than in the shallower fjord, which could contribute to explain the different developments through time for the two communities exposed to AC capping (Fig. S-1c).

The capping treatment with only clay continued to have no negative effects on the benthic community, which confirm the results of no negative effects of a thin deposit of clay in other shorter exposure studies (Näslund et al. 2012; Trannum et al. 2010). In the two deeper reference fields, the number of species was generally lower in Ref-95 than in Ref-80, which probably is an effect of the 15-m difference in depth between the two reference fields. However, Ref-95 is situated in an area that previously has been trawled, and as discussed in Samuelsson et al. (2017), this could also lead to a lower species diversity several years after the disturbance. Trawling can have a negative impact on the number of species, but not always on the species abundance and biomass, depending on the trawling intensity (Skold et al. 2018). Some trawling-sensitive species were found in Ref-95, e.g., the sea urchin *B. lyrifera*, the bivalve *Ennucula tenuis*, and the polychaetes species *Glycera* (Collie et al. 2000; Lindeboom and de Groot 1998), which indicate that the field has recovered from previous trawling. Further, the increase in total abundance after 49 months in Ref-95 was also observed in Ref-80, indicating a natural change in the fjord rather than an effect of recovering from previous trawling activities. This increase in abundance after 49 months was not observed in the same magnitude in AC + clay-95. Besides, the AC field had lower biomass at this time because of no finding of larger organisms such as sea urchins. It appears then that capping with AC + clay also in the deeper area negatively affected the number of species, abundance, and biomass, possibly by preventing the same rate of recruitment as in the reference fields or in other ways act disturbing or harmful for the benthic species.

### Slow succession in the AC fields

The community in the AC field at 30-m depth was still, after more than 4 years, in an intermediate succession stage, with lower abundance, biomass, and number of species compared to the fully developed communities in the reference and clay capped fields. In the deeper fjord, the community in the AC field generally changed in the same direction as the communities in the reference fields, but with an overall reduced recruitment that resulted in a community with fewer and generally smaller species. Succession usually occurs more rapidly in shallow waters than in deeper waters (Diaz and Rosenberg 1995), but in our study under exposure to AC, we have the opposite result. The limited recruitment despite reduced competition and predation from resident adult fauna strengthens our assumption that the slow succession is a result of limited available food resources in the surface sediment layers due to that organic food sources can bind to the AC particles, as discussed in Samuelsson et al. (2017). The slow succession can be compared with an event of bottom water oxygen deficiency during 1997 in the nearby Gullmarsfjord in Sweden, where the benthic fauna was eliminated below 100-m depth and almost depleted below 80-m depth (Rosenberg et al. 2002). Soon after the bottom water was renewed with oxygen rich water, recolonization began and the recovery of the benthic communities was completed, or at least nearly completed, already after 2 years. This recovery occurred even in the deepest parts of the fjord at 118-m. Thus, activated carbon in the sediment seems to have a strong negative effect on the rate of recolonization.

AC can sorb not only organic contaminants but also organic nutriments (Aitcheson et al. 2000; Sessa and Palmquist 2008), which may result in food shortage for the benthic organisms and especially for settling larvae. Many planktonic larvae have the ability to avoid settling in non-suitable habitats and most probably prefer to settle elsewhere than in the AC-capped fields. The strong recruitment of *P. koreni*, with planktotrophic larvae, in the AC field at 30-m depth after 49 months, had been preceded by recruitments of the burrowing bivalves *N. nitidosa* and *Thyasira flexuosa*, both with lecitotrophic larvae. The stored energy in lecitotrophic larvae can facilitate settling in less suitable areas, and their presence can further dilute the active carbon in the sediment surface by bioturbation and thus facilitate settlement of larvae without stored energy, as for example *P. koreni*. Further, AC has in some previous studies shown negative biological effects, e.g., reduced survival, inhibited growth, behavioral changes, reproduction interferences, and morphological disruptions (Janssen et al. 2012; Jonker et al. 2009; Kupryianchyk et al. 2013a, 2011; McLeod et al. 2008; Millward et al. 2005; Nybom et al. 2015, 2012). AC can therefore be disturbing for the benthic species at multiple levels, which together can contribute to the slow succession rate observed here.

### Bioturbation and bioirrigation indices

Both AC fields still had disturbed benthic communities after 49 months, which reduced the sediment reworking processes down to only 11% compared to the references. The bioturbation index BP_c_ was in general higher in the shallower reference fields at 30-m compared to the deeper reference fields at 80–95-m depth. The shallower fjord had a higher presence of species that are known to have high bioturbation and bioirrigation activities, e.g., the brittle star *A. filiformis* and sea urchins (Lohrer et al. 2004; O’Connor et al. 1983; O’Reilly et al. 2006; Solan et al. 2004). The sea urchins are often relatively large, and their presence thus confers high biomass. Besides, the sea urchins are known to have a great influence in the sediment mixing and the associated nutrient and oxygen fluxes in the sediment (Hollertz and Duchene 2001; Lohrer et al. 2004). *A. filiformis* has also a major influence in the sediment mixing (Solan and Kennedy 2002) and can account for up to 80% of the total flux of oxygen into the sediment (Vopel et al. 2003). Further, high abundances of *A. filiformis* are suggested to be of structural importance for the macrofauna community (O’Connor et al. 1983; Rowden et al. 1998; Solan and Kennedy 2002). An extinction of *A. filiformis*, as well as the sea urchins, as in the AC + clay-30 field, therefore has a major impact on the macrofauna community structure and in the sediment mixing and oxygenation, and thus indirectly also on the micro- and meio-fauna communities.

The sea urchins also dominated the biomass in the deeper fjord; however, they were in general fewer but larger compared to 30-m depth. In AC + clay-95, the only finding of a sea urchin was after 14 months. This single sea urchin explains the slightly higher biomass at this time. With their absence after 49 months, the biomass decreased to similar values as after 1 month. Despite the absence of sea urchins, the bioturbation index BP_c_ sustained at the same level after 49 months as after 14 months, due to a slightly higher abundance at this time in combination with the presence of more species that are ranked with higher bioturbation scores, e.g., *T. equalis*, *A. marioni*, and *Abyssoninoe hibernica* (Table S-4b). The bioirrigation indices in the same field did not increase concordantly with the increased abundances from 1 to 49 months or with the temporary biomass increase after 14 months. This was mainly because several important bioirrigators, such as the deposit-feeding polychaetes *H. filiformis*, *L. jeffreysii*, *S. inflatum*, *S. bairdi*, and *Rhodine loveni*, who generally thrive in organic rich environments, had lower abundances in the AC field in the deeper fjord.

The three indices have in common that they combine biomass and abundance, together with species’ scores for either bioturbation or bioirrigation. Using multi-variable indices like this, which summarizes different measured variables in combination with species classifications, can provide a better understanding of the function that the benthic community can sustain. However, it is important to note that the indices only show potential bioturbation and irrigation activity, and not actual measured values. In the context of this article, the indices are used as a measure to quantify loss of function within the reduced benthic communities in the AC fields. The general results from the indices are that the essential functions of bioturbation and bioirrigation in the AC + clay-treated fields were reduced compared to in the reference fields. This highlights a loss of function provided by the benthic organisms that the measured response variables (number of species, abundance, and biomass) might not capture. Further, the reduced function is long-lasting, showing for example the lowest values after 49 months in the deeper AC field. The negative effects would therefore have further ecosystem implications in a larger capped area. Besides, an advantage of thin-layer capping with AC would be that the fauna could survive a thin cap and facilitate the mixing of the sorbent into the sediment through their reworking activity (Ghosh et al. 2011; Sun and Ghosh 2007). The present study has only partly supported this suggestion as the species communities within a 4-year period were clearly affected and therefore the sediment reworking activity was reduced. However, AC was detected down to 5 cm in the sediment, which suggest mixing of the sorbent into the sediment, but this process could have been mainly initial with the presence of bioturbating species such as the brittle star *A. filiformis*.

### Stronger response to activated carbon at 30-m depth

The shallower AC field was in general more negatively affected than the deeper AC field. This could be due to several reasons. Firstly, the number of species was higher at 95-m depth compared to 30-m depth, and the species had a more evenly distributed abundances, i.e., no single species dominated the system as at 30-m depth, giving the deeper ecosystem a higher probability to be more resilient to disturbance (Wittebolle et al. 2009). As the few most abundant sediment reworking species at 30-m depth were severely affected by AC, it had a large influence on the community structure and functioning in terms of bioturbation and bioirrigation.

Secondly, the deeper fjord has approximately three times higher sedimentation rate than the shallower fjord. Besides, the AC + clay slurry that were used in the deeper fjord had slightly lower AC concentration (7% d.w.) compared to the slurry used at 30-m depth (10% d.w.). The concentration of AC in the surface sediment can therefore, in combination with bioturbation, be more rapidly diluted. The calculations of the TOC and AC contents after 49 months showed that the TOC/AC ratio was about four times lower in the shallower fjord compared to the deeper fjord. Lesser amount of sedimentary organic matter, in combination with higher AC content that can sorb all types of organic carbon, will most probably lead to a higher degree of food shortage in the sediment surface at 30-m depth. Consequently, the AC capping may have resulted in reduced potential for how high densities of benthic fauna the sediment can sustain after capping.

Thirdly, some species could suffer more from AC exposure. The community at 30-m depth had higher presence of species that seems to be more vulnerable to AC in this study, for example the echinoderms (brittle stars and sea urchins) together with some polychaetes. The reason why these species are more affected is unclear, but could possibly be coupled to the fine size of AC that was used. Sediment-dwelling organisms without any physical protection (such as shells and tube-living polychaetes) could experience a higher dermal exposure to the powdered AC, e.g., AC particles sticking onto epidermal tissues. The bioturbating community in AC + clay-30 after 49 months consisted for example of 75% of species with physical protection, which implies a loss of species without protection from direct contact with the AC amended sediment. Further, powdered AC could also impede feeding of filter- and deposit-feeding organisms, e.g., either mechanically by clogging cilia and other filtration structures or by decreasing the assimilation of essential nutrients in the gut and thereby cause starvation (Nybom et al. 2015, 2012), and there was a clear reduction in suspension and filter feeding species already after 14 months (Samuelsson et al. 2017). At the same time, powdered AC with particle sizes < 300 μm has proven to be more effective in sorbing contaminants (Kupryianchyk et al. 2011; Rakowska et al. 2012). Remediation with AC has also been proven to be effective when new contaminated sediment particles settle (Cornelissen et al. 2015); however, the presence of deep dwelling benthic organisms is essential to sustain this process since bioturbation can transport AC particles upward into the newly settled material (Abel and Akkanen 2018). The size of the AC particles is therefore a trade-off between lesser negative effects on the benthic macrofauna or higher efficiency in contaminant sequestration.

### Long-term ecosystem effects

The present study is unique in that it describes long-term exposure to AC in natural marine benthic communities over 4 years. It would be hard to mimic the same exposure time in laboratory experiments; however, field experiments do come with some uncertainties, e.g., the settling of larvae and of sedimenting particles can be different between years. It is thus often more difficult in field studies to explain the actual mechanisms behind observed effects, i.e., to explain the reasons behind the reduced species diversity, abundance, and biomass in the AC fields. However, the long-term perspective in this study presents information that AC can be harmful for benthic species for several years. Moreover, reduced bioturbation and bioirrigation capacity in the AC fields would lower the functioning of the ecosystem, e.g., regeneration and circulation of nutrients (Rhoads and Germano 1986; Snelgrove et al. 1997). Besides, less biomass as a consequence of AC may also have negative effects on the ecosystem productivity (Lohrer et al. 2004), as well as causing negative consequences higher up in the trophic food web (Kupryianchyk et al. 2013b). For example, arms of the brittle star *A. filiformis* is an important food source for fish and lobsters (Baden et al. 1990; Duineveld and Vannoort 1986; Mattson 1992), and *A. filiformis* extinction in the AC + clay-30 field would therefore have negative consequences higher up in the food web.

The long-lasting negative effect of powdered AC on the benthic macrofauna in this study needs to be followed up by additional studies in order to observe how long it takes for the benthic community to recover. Further, the mechanisms that are causing these negative biological effects and if they can be reduced by using AC of a larger particle size or another type of sorbent need to be better understood before this type of remediation in situ can be recommended. The contrasting results of biological effects of AC capping in other studies could for example be due to the use of more granular AC or higher TOC contents, as discussed in Samuelsson et al. (2017). For example, only limited negative effects were observed in a fresh water community in Grasse River studied up to a 3-year post-application using activated carbon with a particle size of 75–300 μm and with a TOC content of 4–6% (Beckingham et al. 2013). Further, only minor initial effects and full recovery of benthic organisms after 15 months were observed in a ditch experiment using powdered AC with particle size of 15 μm, but with a TOC of 9.8% (Kupryianchyk et al. 2012). The different environments in combination with the different dose applications of 2–10% AC further complicate the comparisons between studies and their biological effects. It may be that one setup of AC capping could work in one type of environment, but could be devastating in another. Besides, species traits such as feeding type, level of dermal protection, or reproduction and larval development may also need to be considered for a more successful capping with AC. This might be of greater importance if AC capping is considered for a larger area, e.g., an entire fjord branch or even the entire Grenland fjord area.

## Conclusions

The benthic communities at both 30-m and 95-m depths that were exposed to the AC treatment were still severely affected 49 months (i.e., 4 years) after capping. The AC capping resulted in changed communities, especially at 30-m depth, where the ecosystem key species were eradicated. The changes resulted in reduced functioning of the benthic community; only 11–44% of the calculated potential bioturbation and bioirrigation activities remained in the AC fields after 49 months. This highlights the importance of field experiments, where long-term effects can be evaluated under natural conditions. The substantial community changes and connecting ecosystem consequences should be carefully considered in remediation with powdered AC, since the results presented in this study show severe long-lasting disturbances that have not been previously reported.

## Supplementary information

ESM 1 (PDF 1273 kb)

## Data Availability

All data generated or analyzed during this study are included in this published article and its supplementary information files.
