# Supplementary material for: Impaired benthic macrofauna function 4 years after sediment capping with activated carbon in the Grenland fjords, Norway
Source: Environ Sci Pollut Res Int. 2020 Dec 2;28(13):16181–97. doi: 10.1007/s11356-020-11607-0 (PMC7969561; doi:10.1007/s11356-020-11607-0)
Supplement: Supplementary file 1 — (PDF 1273 kb) [file 11356_2020_11607_MOESM1_ESM.pdf]

Supplementary material

Impaired benthic macrofauna function four years after sediment capping with activated carbon in the Grenland fjords, Norway

Caroline Raymond[1]*, Göran S Samuelsson[2], Stefan Agrenius[3], Morten Schaanning[4], Jonas S Gunnarsson[1]

[1] Department of Ecology, Environment and Plant Sciences (DEEP), Stockholm University, 106 91 Stockholm, Sweden,
[2] Svensk Ekologikonsult, Skiftesvägen 17, 163 43 Stockholm, Sweden
[3] Department of Marine Sciences–Kristineberg, University of Gothenburg, 451 78 Fiskebäckskil, Sweden
[4] Norwegian Institute for Water Research (NIVA), 0349 Oslo, Norway

*Corresponding author: caroline.raymond@su.se

Table S-1 (a-f). Species list with abundance and biomass per grab

Table S-2. Species classifications

Figure S-1 (a-c). Additional biometrics

Table S-3. PERMANOVA main test

Table S-4 (a-d). Most important species in each field after 1, 14 and 49 months

Number of pages: 21

Table S-1. Species list. Abundance (Abu) per grab and Biomass (Bio) g wet weight per grab.

a. AC+clay-30

Phylum	Taxon	AC+clay-30:1A		AC+clay-30:1B		AC+clay-30:1C		AC+clay-30:14A		AC+clay-30:14B		AC+clay-30:14C		AC+clay-30:14D		AC+clay-30:14E		AC+clay-30:49A		AC+clay-30:49B		AC+clay-30:49C		AC+clay-30:49D		AC+clay-30:49E	
		Abu	Bio	Abu	Bio	Abu	Bio	Abu	Bio	Abu	Bio	Abu	Bio	Abu	Bio	Abu	Bio	Abu	Bio	Abu	Bio	Abu	Bio	Abu	Bio	Abu	Bio
Annelida	*Ampharete octocirrata*																			1	0.01						
Annelida	*Heteromastus filiformis*	1	0.01	1	0.005																	3	0.01	4	0.005	1	0.01
Annelida	*Notomastus latericeus*									1	0.14													1	0.005		
Annelida	*Chaetopterus norvegicus*															1	1.11										
Annelida	*Chaetozone setosa*																	1	0.01			2	0.01				
Annelida	*Cossura longocirrata*	1	0.005																								
Annelida	*Diplocirrus glaucus*	1	0.01																			3	0.01			1	0.01
Annelida	*Glycera alba*															1	0.005										
Annelida	*Goniada maculata*															1	0.02			1	0.01					1	0.11
Annelida	*Abyssoninoe hibernica*	1	0.02													5	0.12	1	0.02	1	0.04						
Annelida	*Magelona filiformis*																	1	0.01	1	0.01	2	0	1	0.005		
Annelida	*Nephtys incisa*	3	0.25	4	0.33			2	0.06	2	0.19							5	0.38	4	0.28	2	0.16	2	0.34	1	0.12
Annelida	*Levinsenia gracilis*																							1	0.005		
Annelida	*Pectinaria koreni*																	29	0.1	11	0.78	10	0.21	9	0.62	59	1.66
Annelida	*Bylgides elegans*																							1	0.005		
Annelida	*Scalibregma inflatum*	1	0.01	1	0.03	2	0.18							2	0.03												
Annelida	*Polydora* spp.																	1	0.01								
Annelida	*Spiophanes kroeyeri*							1	0.01	1	0.03																
Annelida	*Polycirrus* spp.	2	0.01			1	0.01																				
Annelida	*Trichobranchus roseus*	1	0.01	2	0.03	1	0.03																				
Arthropoda	*Callianassa subterranea*					1	0.02																				
Echinodermata	*Amphiura filiformis*			1	0.005	1	0.005	1	0.02																		
Echinodermata	*Marthasterias glacialis cf.*																					1	0.01				
Echinodermata	*Brissopsis lyrifera*	1	8.79			1	9.25									1	8.84										
Echinodermata	*Leptopentacta elongata*																							1	0.2		
Echinodermata	*Echinocardium cordatum*	2	8.58	1	3.24	1	5.3																				
Echinodermata	*Luidia sarsi*	2	0.01			1	0.005																				
Mollusca	*Parvicardium minimum*	1	0.005																								
Mollusca	*Corbula gibba*	3	0.73	1	0.005	1	0.33									4	0.31			2	0.06			2	0.19		
Mollusca	*Cylichna cylindracea*	2	0.02	1	0.01	5	0.05			1	0.02																
Mollusca	*Hyala vitrea*	5	0.02	2	0.01	2	0.01											1	0.01								
Mollusca	*Tellimya ferruginosa*	3	0.005	1	0.005	1	0.005																				
Mollusca	*Tellimya tenella*	2	0.01			3	0.01																				
Mollusca	*Euspira nitida*	1	0.03	1	0.005																						
Mollusca	*Nucula nitidosa*	1	0.79															13	0.46	6	0.18	7	0.12	11	0.31	15	0.38
Mollusca	*Hermania scabra*			1	0.01									2	0.05			3	0.04	1	0.02	2	0.02	1	0.01	1	0.01
Mollusca	*Abra nitida*	1	0.005	2	0.04							1	0.08					6	1.35			4	0.63	2	0.44	1	0.12
Mollusca	*Thyasira flexuosa*																	4	0.08	3	0.04	3	0.07	3	0.1	1	0.03
Mollusca	*Thyasira sarsii*											1	0.01	1	0.01	1	0.01										
Nemertea	Nemertea																	1	0.09							2	0.01
Nemertea	*Cerebratulus spp.*											1	0.02														
Sipuncula	*Golfingia vulgaris*									1	0.01																
Sipuncula	*Thysanocardia procera*																			1	0.09						

b. Clay-30

Phylum	Taxon	Clay-30:1A		Clay-30:1B		Clay-30:1C		Clay-30:14A		Clay-30:14B		Clay-30:14C		Clay-30:14D		Clay-30:14E		Clay-30:49A		Clay-30:49B		Clay-30:49C		Clay-30:49D		Clay-30:49E	
		Abu	Bio	Abu	Bio	Abu	Bio	Abu	Bio	Abu	Bio	Abu	Bio	Abu	Bio	Abu	Bio	Abu	Bio	Abu	Bio	Abu	Bio	Abu	Bio	Abu	Bio
Annelida	*Ampharete finmarchica*									4	0.01																
Annelida	*Heteromastus filiformis*	1	0.005													1	0.005	1	0.02								
Annelida	*Chaetopterus norvegicus*															1	0.12							1	0.16		
Annelida	*Spiochaetopterus typicus*																	1	0.24								
Annelida	*Chaetozone setosa*															1	0.005	3	0.03	1	0.01			3	0.02	2	0.01
Annelida	*Brada villosa*																					1	0.15				
Annelida	*Diplocirrus glaucus*	4	0.03			3	0.02	1	0.01	3	0.005	1	0.005	2	0.02			2	0.02	4	0.03	4	0.03			2	0.01
Annelida	*Glycera alba*			1	0.01			3	0.07			1	0.005	1	0.02	1	0.02	2	0.09	2	0.07			1	0.04	1	0.04
Annelida	*Glycera unicornis*	1	0.08							1	0.22																
Annelida	*Goniada maculata*													2	0.12			3	0.03	2	0.02	2	0.04			1	0.02
Annelida	*Oxydromus flexuosus*													1	0.005									1	0.06		
Annelida	*Psamathe fusca*													1	0.005												
Annelida	*Abyssoninoe hibernica*	3	0.06	1	0.01	8	0.21	1	0.03	1	0.02	1	0.04			1	0.005	3	0.05	2	0.08	3	0.14	3	0.08	2	0.07
Annelida	*Magelona filiformis*																	1	0.01								
Annelida	*Praxillella affinis*																	1	0.06	1	0.03	2	0.07	1	0.02		
Annelida	*Praxillella praetermissa*	1	0.03																								
Annelida	*Rhodine gracilior*																			1	0.21						
Annelida	*Nephtys incisa*					1	0.07	3	0.38	2	0.29	2	0.24	2	0.35	1	0.18	1	0.06	1	0.01			5	0.16	1	0.06
Annelida	*Galathowenia oculata*																			1	0.01						
Annelida	*Pectinaria auricoma*	1	0.01	3	0.16															1	0.03						
Annelida	*Pectinaria belgica*	2	0.005			2	1.53							1	0.44					3	1.72	2	3.95	1	0.005	11	0.17
Annelida	*Pectinaria koreni*											2	0.13														
Annelida	*Phyllodoce groenlandica*							1	0.005															1	0.005		
Annelida	*Eunoe nodosa*											1	0.005														
Annelida	*Gattyana cirrhosa*											2	0.005														
Annelida	*Lipobranchius jeffreysii*																					1	0.53				
Annelida	*Polyphysia crassa*			3	0.84	1	0.9																				
Annelida	*Scalibregma inflatum*	1	0.005					53	0.28	18	0.1	21	0.08	6	0.03	39	0.14	3	0.02			1	0.01	5	0.04	1	0.01
Annelida	*Pholoe baltica*																	1	0.01	2	0.01					1	0.01
Annelida	*Laonice bahusiensis*	1	0.01																								
Annelida	*Polydora* spp.	1	0.005					9	0.005			2	0.005														
Annelida	*Prionospio dubia*																	1	0.03								
Annelida	*Prionospio fallax*									3	0.005																
Annelida	*Spiophanes kroeyeri*	1	0.04	2	0.01	1	0.01			2	0.04	1	0.05					2	0.02	2	0.03	1	0.01			1	0.01
Annelida	*Polycirrus* spp.																	2	0.03	2	0.01	1	0.01	1	0.005	1	0.07
Annelida	*Terebellides stroemi*	3	0.03							1	0.01					4	0.02	1	0.02							1	0.03
Annelida	*Trichobranchus roseus*											1	0.02							3	0.02	2	0.04	1	0.02	1	0.04
Arthropoda	*Callianassa subterranea*	1	0.005	1	0.01	1	0.01											2	0.03					1	0.005	2	0.06
Arthropoda	*Diastylis boecki*																	1	0.01								
Arthropoda	*Leucothoe lilljeborgii*																							1	0.005		
Arthropoda	*Eriopisa elongata*					1	0.005			1	0.005									1	0.01	2	0.01	2	0.005		
Cnidaria	Edwardsiidae			1	0.01	1	0.005			4	0.06	1	0.02	1	0.005					1	0.01						
Echinodermata	*Amphiura chiajei*	2	0.39	4	0.596	1	0.303			4	0.877			4	1.205			3	0.26	3	0.32	3	0.2	4	0.29	7	0.71
Echinodermata	*Amphiura filiformis*	23	0.66	41	1.944	24	1.348	18	0.32	21	1.523	27	1.15	29	2.145	8	0.33	52	3.95	74	4.82	71	6.21	102	8.23	39	3.69
Echinodermata	*Brissopsis lyrifera*	2	25.51			2	17.49	1	4.71	1	6.07	1	7.52	1	6.48	1	2.85			1	9.73	2	17.8			2	8.53
Echinodermata	*Leptopentacta elongata*			1	0.03					1	0.49																
Echinodermata	*Echinocardium cordatum*					2	5.3					3	9.82	2	7.95												
Echinodermata	*Echinocardium flavescens*			1	2.99													1	5.32	1	0.53	1	2.53			1	2.61

Echinodermata	*Luidia sarsi*	1	0.005																								
Echinodermata	*Mesothuria intestinalis*											1	34.7														
Echinodermata	*Labidoplax buskii*																							1	0.005		
Mollusca	*Chaetoderma nitidulum*	1	0.03	1	0.06							2	0.14														
Mollusca	*Corbula gibba*	7	1	13	0.5	10	0.63	6	0.54	6	0.43	9	0.5	5	0.05	2	0.33	1	0.05	6	0.65			3	0.02	6	0.35
Mollusca	*Cuspidaria obesa*					1	0.08																				
Mollusca	*Cylichna cylindracea*					3	0.05			1	0.02									2	0.03					4	0.04
Mollusca	*Antalis entalis*					1	0.68															1	0.36				
Mollusca	*Hyala vitrea*	19	0.05	2	0.01	4	0.01			3	0.005	1	0.005			1	0.005	1	0.01	1	0.01	4	0.01				
Mollusca	*Kurtiella bidentata*					2	0.005																				
Mollusca	*Tellimya ferruginosa*																	1	0.01								
Mollusca	*Tellimya tenella*					3	0.005																				
Mollusca	*Myrtea spinifera*											1	0.03														
Mollusca	*Euspira nitida*					1	0.15					1	0.14			1	0.07										
Mollusca	*Ennucula tenuis*													1	0.08												
Mollusca	*Nucula nitidosa*	13	0.35	8	0.27	2	0.09					2	0.03	3	0.17	2	0.14							1	0.005	2	0.04
Mollusca	*Phaxas pellucidus*											2	0.03														
Mollusca	*Hermania scabra*	1	0.01			3	0.05	1	0.06	1	0.01	1	0.005					1	0.01	3	0.03	2	0.02	7	0.08	3	0.07
Mollusca	*Abra nitida*					1	0.01					1	0.06							1	0.01	1	0.01	2	0.005	4	0.05
Mollusca	*Thyasira flexuosa*	4	0.03	5	0.02			7	0.21			3	0.08									2	0.01			1	0.01
Mollusca	*Thyasira sarsii*													1	0.005												
Nemertea	*Cerebratulus* spp.													3	9.78					1	2.23			1	0.05		
Sipuncula	*Golfingia vulgaris*									1	0.17																
Sipuncula	*Thysanocardia procera*	1	0.06			4	0.24	1	0.15	1	0.05					1	0.06					1	0.03			1	0.04

c. Ref-30

		Ref-30:1A		Ref-30:1B		Ref-30:1C		Ref-30:14A		Ref-30:14B		Ref-30:14C		Ref-30:14D		Ref-30:14E		Ref-30:49A		Ref-30:49B		Ref-30:49C		Ref-30:49D		Ref-30:49E	
**Phylum**	**Taxon**	**Abu**	**Bio**	**Abu**	**Bio**	**Abu**	**Bio**	**Abu**	**Bio**	**Abu**	**Bio**	**Abu**	**Bio**	**Abu**	**Bio**	**Abu**	**Bio**	**Abu**	**Bio**	**Abu**	**Bio**	**Abu**	**Bio**	**Abu**	**Bio**	**Abu**	**Bio**
Annelida	*Sosane sulcata*																	1	0.03								
Annelida	*Heteromastus filiformis*									1	0.02	2	0.01					1	0.01								
Annelida	*Chaetopterus norvegicus*									1	1.37																
Annelida	*Chaetozone setosa*							1	0.005											1	0.01	2	0.01	3	0.02	1	0.01
Annelida	*Diplocirrus glaucus*	1	0.01	1	0.01			4	0.04	5	0.04	2	0.005	2	0.005	2	0.005	3	0.01			2	0.03	3	0.03	6	0.05
Annelida	*Glycera alba*							1	0.05	1	0.01			2	0.07			1	0.01	1	0.08	1	0.08			1	0.01
Annelida	*Goniada maculata*			1	0.06	1	0.07	1	0.02									1	0.01	2	0.08	1	0.02	2	0.05	3	0.05
Annelida	*Oxydromus flexuosus*							1	0.04	2	0.03	1	0.02	2	0.06	2	0.04					1	0.02				
Annelida	*Podarkeopsis helgolandicus*					1	0.01															1	0.03				
Annelida	*Abyssoninoe hibernica*			4	0.08	1	0.03	3	0.1	3	0.07			2	0.09			6	0.11	3	0.12	5	0.12	4	0.15	2	0.04
Annelida	*Maldanidae* indet.									1	0.02																
Annelida	*Praxillella affinis*													2	0.07			3	0.15	3	0.2	3	0.12	2	0.09	8	0.3
Annelida	*Praxillella praetermissa*	2	0.07					3	0.09							2	0.07										
Annelida	*Nephtys incisa*	1	0.09	1	0.02	1	0.09	1	0.07	1	0.005	2	0.17	1	0.16	1	0.08	1	0.06	1	0.07	1	0.01	1	0.02	4	0.12
Annelida	*Galathowenia oculata*																									1	0.01
Annelida	*Owenia fusiformis*																					1	0.01				
Annelida	*Pectinaria auricoma*									1	0.005	2	0.01											1	0.02		
Annelida	*Pectinaria belgica*							4	4.29			1	0.005							1	0.63						
Annelida	*Bylgides elegans*																			1	0.01						
Annelida	*Lipobranchius jeffreysii*	1	0.82																	1	0.64						
Annelida	*Scalibregma inflatum*							23	0.09	56	0.21	10	0.12	35	0.53	8	0.05	1	0.03	1	0.01			4	0.05	1	0.01
Annelida	*Pholoe baltica*											1	0.005	1	0.005	1	0.01	1	0.01								

Annelida	*Polydora* spp.							1	0.005																		
Annelida	*Prionospio fallax*																									1	0.01
Annelida	*Scolelepis tridentata*									1	0.005															2	0.01
Annelida	*Spiophanes kroeyeri*	2	0.05			2	0.03	2	0.03	2	0.06	2	0.03	2	0.03	3	0.06			1	0.01	1	0.01			1	0.01
Annelida	*Polycirrus* spp.	1	0.02															1	0.01							1	0.08
Annelida	*Streblosoma bairdi*									1	0.05	1	0.05	1	0.07	2	0.01										
Annelida	*Terebellides stroemi*			1	0.01					6	0.03			8	0.09	1	0.02	1	0.01	4	0.04	1	0.01	2	0.03	4	0.06
Annelida	*Trichobranchus roseus*																									1	0.02
Arthropoda	*Ampelisca tenuicornis*																							1	0.005	1	0.01
Arthropoda	*Callianassa subterranea*	1	0.12	2	0.04	1	0.02			1	0.01	3	0.12	3	0.08	3	0.13							4	0.55	3	0.06
Arthropoda	*Diastylis laevis*															1	0.02										
Arthropoda	*Eriopisa elongata*	3	0.01							1	0.005																
Arthropoda	Oedicerotidae									1	0.005																
Cnidaria	Edwardsiidae											1	0.005			1	0.005	1	0.02							2	0.02
Echinodermata	*Amphiura chiajei*	1	0.063	1	0.136	1	0.024											3	0.19	4	0.25	2	0.19	1	0.15	1	0.06
Echinodermata	*Amphiura filiformis*	24	0.907	24	1.634	17	0.496	8	0.24	7	0.1	3	0.09	9	0.59	14	0.5	59	6.64	47	4.35	47	3.41	28	2.61	36	2.33
Echinodermata	*Brissopsis lyrifera*	1	3.18	1	7.89	2	11.07							1	7.69	1	14.72	4	31.6	3	25.4			2	11.03	3	32.8
Echinodermata	*Leptopentacta elongata*																			1	0.01					1	0.56
Echinodermata	*Echinocardium cordatum*			1	5.36			1	3.95							2	7.12										
Echinodermata	*Echinocardium flavescens*																	3	1.67	2	1.04			15	15.97	5	11.3
Echinodermata	*Luidia sarsi*	1	0.01																								
Echinodermata	*Labidoplax buskii*																									1	0.05
Mollusca	*Parvicardium minimum*																									1	0.05
Mollusca	*Parvicardium pinnulatum*													1	0.05	1	0.07										
Mollusca	*Chaetoderma nitidulum*			1	0.07	1	0.05																	1	0.03		
Mollusca	*Corbula gibba*	12	0.61	3	0.58	3	0.19			1	0.005									1	0.29			1	0.04		
Mollusca	*Cylichna cylindracea*	4	0.05			3	0.04									2	0.03					1	0.02				
Mollusca	*Antalis entalis*																									1	1.51
Mollusca	*Hyala vitrea*	2	0.005			2	0.01	1	0.005							1	0.005										
Mollusca	*Kurtiella bidentata*																									1	0.01
Mollusca	*Nucula nitidosa*	2	0.02					1	0.07			1	0.08							2	0.01					1	0.01
Mollusca	*Hermania scabra*					2	0.01			1	0.01					1	0.04			2	0.03	1	0.01	1	0.01		
Mollusca	*Abra alba*																							1	0.005		
Mollusca	*Abra nitida*	1	0.04	1	0.06																	1	0.01	1	0.02	1	0.01
Mollusca	*Thyasira flexuosa*											1	0.005									2	0.01	2	0.01	2	0.01
Mollusca	*Mysia undata*							1	0.12																		
Nemertea	Nemertea					1	1.42			1	0.72	1	0.02	1	0.09									1	0.005		
Nemertea	*Cerebratulus* spp.																					1	6.13				
Sipuncula	*Golfingia vulgaris*																			1	0.64						
Sipuncula	*Thysanocardia procera*	2	0.22	1	0.09							1	0.12	2	0.17	1	0.1					1	0.02	1	0.12	2	0.09

d. AC+clay-95

		AC+clay-95:1A		AC+clay-95:1B		AC+clay-95:1C		AC+clay-95:14A		AC+clay-95:14B		AC+clay-95:14C		AC+clay-95:14D		AC+clay-95:14E		AC+clay-95:49A		AC+clay-95:49B		AC+clay-95:49C		AC+clay-95:49D		AC+clay-95:49E	
**Phylum**	**Taxon**	**Abu**	**Bio**	**Abu**	**Bio**	**Abu**	**Bio**	**Abu**	**Bio**	**Abu**	**Bio**	**Abu**	**Bio**	**Abu**	**Bio**	**Abu**	**Bio**	**Abu**	**Bio**	**Abu**	**Bio**	**Abu**	**Bio**	**Abu**	**Bio**	**Abu**	**Bio**
Annelida	*Melinna cristata*	2	0.03																			1	0.04				
Annelida	*Paramphinome jeffreysi*	1	0.01	6	0.03	3	0.02			56	0.21	24	0.07	9	0.03	55	0.2	40	0.17	19	0.09	11	0.04	17	0.07	25	0.11
Annelida	*Heteromastus filiformis*	2	0.01			2	0.005	4	0.01	4	0.005	4	0.01	1	0.005	3	0.005	3	0.01	12	0.03	8	0.02	7	0.01	5	0.01
Annelida	*Aphelochaeta* sp.							1	0.005	1	0.005																
Annelida	*Aphelochaeta marioni*	2	0.02	4	0.03	1	0.02	3	0.02	4	0.04	3	0.02	6	0.16	4	0.04	23	0.35	20	0.2	6	0.06	3	0.01	9	0.08
Annelida	*Chaetozone setosa*	2	0.01	10	0.08			1	0.005	5	0.005	4	0.02	2	0.02	4	0.02	5	0.01	2	0.01	2	0.01	1	0.005	1	0.01

Annelida	*Diplocirrus glaucus*			1	0.01	1	0.01			1	0.005					1	0.005										
Annelida	*Glycera alba*			1	0.02			2	0.09	2	0.06	3	0.18	1	0.005	4	0.29			1	0.01	2	0.15	1	0.03		
Annelida	*Glycera unicornis*											1	0.53									1	0.69				
Annelida	*Glycinde nordmanni*											1	0.02							1	0.01			1	0.005		
Annelida	*Goniada maculata*									1	0.005			1	0.03	1	0.02	1	0.01								
Annelida	*Oxydromus flexuosus*							1	0.04																		
Annelida	*Abyssoninoe* sp.																					1	0.17				
Annelida	*Abyssoninoe hibernica*			1	0.09													1	0.06	2	0.05	2	0.04	1	0.02	3	0.08
Annelida	*Scoletoma fragilis*													1	0.24												
Annelida	*Praxillella affinis*	1	0.005																								
Annelida	*Rhodine loveni*	2	0.18			1	0.03	2	0.09			1	0.02			2	0.04										
Annelida	*Nephtys incisa*															1	0.005	1	0.01	1	0.06						
Annelida	*Nephtys paradoxa*																							1	0.09		
Annelida	*Ceratocephale loveni*	2	0.05	5	0.12	1	0.01	4	0.05	3	0.06	3	0.02	1	0.005	2	0.03			2	0.01						
Annelida	*Phylo norvegica*					1	0.37																				
Annelida	*Galathowenia oculata*									1	0.005			1	0.005			1	0.01	1	0.01						
Annelida	*Chaetoparia nilssoni*	1	0.005									1	0.01							1	0.01						
Annelida	*Phyllodoce groenlandica*																			1	0.01	1	0.01			1	0.01
Annelida	*Phyllodoce rosea*					1	0.01																				
Annelida	*Bylgides elegans*											2	0.02														
Annelida	*Gattyana amondseni*											1	0.01														
Annelida	*Harmothoe* sp.											1	0.01														
Annelida	Polynoidae									2	0.005			1	0.005	1	0.005										
Annelida	*Euchone papillosa*					2	0.02	1	0.005	2	0.01					1	0.005			2	0.01			2	0.005		
Annelida	*Scalibregma inflatum*			2	0.06	1	0.02					1	0.01					2	0.02			1	0.02	1	0.005	1	0.01
Annelida	*Pholoe baltica*																							1	0.005		
Annelida	*Polydora* spp.													1	0.005												
Annelida	*Prionospio cirrifera*																	17	0.02	21	0.04	19	0.06	13	0.03	15	0.03
Annelida	*Prionospio dubia*	1	0.01	1	0.005																						
Annelida	*Prionospio fallax*																			4	0.01						
Annelida	*Spiophanes kroeyeri*	7	0.16	7	0.1	10	0.13	5	0.05	14	0.09	9	0.04	2	0.02	15	0.16	12	0.1	13	0.12	5	0.04	13	0.11	10	0.09
Annelida	*Neoamphitrite affinis*	1	0.01																	1	0.07						
Annelida	*Neoamphitrite grayi*	1	0.61																								
Annelida	*Pista cristata*	1	0.07	2	0.28			1	0.03																		
Annelida	*Terebellides stroemi*					1	0.005																				
Arthropoda	Tanaidacea indet.	2	0.005			1	0.005																				
Arthropoda	*Diastylis boecki*			1	0.01																						
Arthropoda	*Diastylis cornuta*																			2	0.01					1	0.01
Arthropoda	*Diastyloides serratus*					6	0.01					1	0.005														
Arthropoda	*Leptostylis longimana*					1	0.005																				
Arthropoda	*Gnathia oxyuraea*			1	0.005																						
Arthropoda	*Eudorella emarginata*							1	0.005	3	0.005			3	0.02	2	0.005	4	0.01	7	0.02	1	0.01	4	0.01	3	0.01
Arthropoda	*Eudorella truncatula*																			2	0.01					1	0.01
Arthropoda	*Leucon nasica*					1	0.005	1	0.005	2	0.005			2	0.01	2	0.005	6	0.03	11	0.05	5	0.01	8	0.02	8	0.02
Arthropoda	*Hippomedon propinquus*																	1	0.01	1	0.01						
Arthropoda	Lysianassidae									1	0.005																
Arthropoda	*Campylaspis costata*					1	0.005																				
Arthropoda	*Arrhis phyllonyx*							1	0.02			3	0.07	6	0.14	3	0.06	2	0.02			1	0.02			3	0.04
Arthropoda	*Monoculodes carinatus*									1	0.005																
Arthropoda	*Westwoodilla caecula*																			4	0.02	1	0.01			2	0.01
Arthropoda	*Philomedes brenda*	2	0.005			4	0.01					1	0.005					1	0.01			1	0.01	2	0.005	6	0.01
Echinodermata	*Amphiura chiajei*							1	0.008									1	0.17	1	0.25			2	0.01		
Echinodermata	*Amphiura filiformis*					1	0.005	1	0.008													1	0.04				

Echinodermata	*Brissopsis lyrifera*							1	12.11																		
Echinodermata	*Ophiocten affinis*					1	0.08																				
Echinodermata	*Ophiura albida*																	1	0.01								
Mollusca	Nudibranchia									1	0.02																
Mollusca	*Parvicardium pinnulatum*							1	0.02																		
Mollusca	*Corbula gibba*	1	0.01																								
Mollusca	*Tropidomya abbreviata*																									1	0.05
Mollusca	*Hyala vitrea*	3	0.01																								
Mollusca	*Kurtiella bidentata*	1	0.005																								
Mollusca	*Ennucula tenuis*	1	0.005																	2	0.04						
Mollusca	*Hermania scabra*							3	0.05	3	0.03	4	0.06	1	0.02	1	0.03	1	0.01	2	0.01	1	0.04				
Mollusca	*Philine quadripartita*																	1	0.03			2	0.01	1	0.005		
Mollusca	*Abra nitida*	1	0.005	1	0.01	3	0.005	13	0.55	2	0.08	9	0.23	13	0.38	13	0.37			1	0.09			1	0.09		
Mollusca	*Thyasira equalis*	22	0.43	28	0.34	18	0.18	1	0.05	17	0.5	27	0.54	3	0.09	12	0.39	29	0.43	21	0.51	17	0.36	20	0.59	22	0.39
Mollusca	*Thyasira sarsii*					1	0.005											1	0.03			2	0.1				
Mollusca	*Yoldiella philippiana*	10	0.13	5	0.04	12	0.17	1	0.02	4	0.06	4	0.03	2	0.05			3	0.05	5	0.11	4	0.08	3	0.06	2	0.04
Nemertea	Nemertea																	1	0.01	1	0.01	2	0.09			1	0.01
Nemertea	*Cerebratulus* spp.							1	0.04																		
Platyhelminthes	Turbellaria											1	0.03											1	0.27		
Sipuncula	*Phascolion strombus*	6	0.005	5	0.005	1	0.005																				

e. Ref-80

		Ref-80:1A		Ref-80:1B		Ref-80:1C		Ref-80:14A		Ref-80:14B		Ref-80:14C		Ref-80:14D		Ref-80:14E		Ref-80:49A		Ref-80:49B		Ref-80:49C		Ref-80:49D		Ref-80:49E	
**Phylum**	**Taxon**	**Abu**	**Bio**	**Abu**	**Bio**	**Abu**	**Bio**	**Abu**	**Bio**	**Abu**	**Bio**	**Abu**	**Bio**	**Abu**	**Bio**	**Abu**	**Bio**	**Abu**	**Bio**	**Abu**	**Bio**	**Abu**	**Bio**	**Abu**	**Bio**	**Abu**	**Bio**
Annelida	*Anobothrus gracilis*											1	0.01			1	0.005					1	0.02				
Annelida	*Eclysippe eliasoni*	2	0.005			1	0.01	1	0.005	2	0.01	3	0.005	1	0.005	1	0.005					4	0.01	2	0.005	4	0.01
Annelida	*Melinna cristata*	3	0.03					1	0.03											1	0.12					1	0.04
Annelida	*Mugga wahrbergi*																					5	0.01				
Annelida	*Sosane sulcata*	1	0.01			1	0.005											2	0.02			3	0.01			2	0.01
Annelida	*Paramphinome jeffreysi*			3	0.02	3	0.01	13	0.04	33	0.13	14	0.05	46	0.15	20	0.06	7	0.04	2	0.01	11	0.04	8	0.014	3	0.02
Annelida	*Heteromastus filiformis*	4	0.02	3	0.01	1	0.005	7	0.02	11	0.03	9	0.02	5	0.01	8	0.02	39	0.17	21	0.14	25	0.12	8	0.033	19	0.12
Annelida	*Notomastus latericeus*	1	0.06	1	0.03																					2	0.15
Annelida	*Aphelochaeta marioni*	4	0.04	2	0.02	4	0.04	2	0.01	9	0.09	15	0.13	20	0.25	11	0.15	11	0.11	8	0.09	11	0.07	9	0.133	6	0.09
Annelida	*Chaetozone setosa*	1	0.01			1	0.005	13	0.07	1	0.005	12	0.05	7	0.04	11	0.05	38	0.18	22	0.1	18	0.09	16	0.09	4	0.03
Annelida	*Tharyx killariensis*			1	0.01																						
Annelida	*Iphitime hartmanae*	1	0.08																								
Annelida	*Brada villosa*									2	0.01	1	0.005	2	0.07	6	0.18	3	0.06					1	0.033		
Annelida	*Diplocirrus glaucus*	2	0.02	3	0.01	1	0.01	1	0.01	5	0.03	1	0.01	1	0.01	3	0.01	8	0.11	3	0.04	9	0.07	1	0.008	10	0.08
Annelida	*Glycera alba*	1	0.02			4	0.16	2	0.03	3	0.08	4	0.12	3	0.06	3	0.02			2	0.26	2	0.02	3	0.064	5	0.2
Annelida	*Glycera unicornis*	2	0.38							2	0.47	1	1.08														
Annelida	*Glycinde nordmanni*					1	0.01																	1	0.032		
Annelida	*Goniada maculata*							1	0.04	1	0.05			2	0.07	1	0.01	1	0.01	1	0.01	1	0.03	3	0.05		
Annelida	*Oxydromus flexuosus*									1	0.005	2	0.04			1	0.01	1	0.02								
Annelida	*Abyssoninoe hibernica*	3	0.12	4	0.13	2	0.07	3	0.07	2	0.03	5	0.16	6	0.42	2	0.08	3	0.11	1	0.03	5	0.09	4	0.158	1	0.01
Annelida	*Lumbrineris gracilis*																									1	0.01
Annelida	*Scoletoma fragilis*			1	0.52																			1	0.222	1	0.13
Annelida	*Scoletoma impatiens*																									1	0.04
Annelida	*Maldane sarsi*			3	0.01																						
Annelida	*Praxillella affinis*	1	0.005	1	0.005													2	0.02			6	0.08			2	0.03
Annelida	*Praxillella praetermissa*																							1	0.063		

Annelida	*Rhodine loveni*			1	0.09	1	0.01	3	0.24	2	0.38	4	0.08			1	0.06	1	0.16	4	0.1	6	0.29	1	0.132	3	0.09
Annelida	*Nephtys incisa*																	1	0.05								
Annelida	*Ceratocephale loveni*					3	0.03	4	0.05	2	0.04	4	0.04	3	0.04	2	0.005	5	0.04	3	0.09	2	0.05	2	0.045		
Annelida	*Drilonereis filum*																			1	0.5						
Annelida	*Ophelina* sp.																					1	0.01				
Annelida	*Ophelina norvegica*															1	0.02	1	0.03								
Annelida	*Galathowenia oculata*					1	0.005					3	0.02					2	0.01			6	0.01			1	0.01
Annelida	*Levinsenia gracilis*																					5	0.01			3	0.01
Annelida	*Pectinaria belgica*			1	1.67																						
Annelida	*Pectinaria koreni*									1	0.01			1	0.04												
Annelida	*Eteone* sp.																							1	5E-04		
Annelida	*Eteone longa* cf.																					1	0.01				
Annelida	*Eumida bahusiensis*													1	0.005												
Annelida	*Nereiphylla lutea*	1	0.01									1	0.005														
Annelida	*Phyllodoce groenlandica*																			1	0.01					1	0.01
Annelida	*Phyllodoce rosea*			1	0.005																			1	0.005		
Annelida	Phyllodocidae											1	0.005														
Annelida	*Sige fusigera*																	1	0.01								
Annelida	*Glyphohesione klatti*			1	0.01																						
Annelida	*Bylgides elegans*									1	0.005							2	0.01	2	0.02	1	0.01	3	0.003	1	0.01
Annelida	*Gattyana amondseni*			1	0.005																						
Annelida	*Harmothoe* sp.							1	0.005																		
Annelida	*Harmothoe borealis* cf.																					2	0.01			1	0.01
Annelida	Polynoidae	1	0.005	1	0.005			2	0.01			1	0.005														
Annelida	*Euchone papillosa*			1	0.005	1	0.02	2	0.005							1	0.005					1	0.01			1	0.01
Annelida	*Jasmineira caudata*																					3	0.01				
Annelida	*Lipobranchius jeffreysii*			2	1.08			6	1.64	2	0.53	5	1.4	3	1.62	5	1.2	3	1.06	3	1.85	1	0.2			1	0.55
Annelida	*Polyphysia crassa*																							1	0.508		
Annelida	*Scalibregma inflatum*							2	0.12	3	0.01	5	0.03	4	0.01	2	0.02	22	0.45	20	0.32	14	0.15	14	0.325	2	0.04
Annelida	*Pholoe baltica*							1	0.005	1	0.005							1	0.01			4	0.01	2	0.003	1	0.01
Annelida	*Pholoe pallida*							1	0.005									2	0.01					1	0.006	1	0.01
Annelida	*Laonice bahusiensis*																					1	0.01				
Annelida	*Polydora* spp.															1	0.005					7	0.01			4	0.01
Annelida	*Prionospio cirrifera*							1	0.005	1	0.005	1	0.005					50	0.07	3	0.01	25	0.06	18	0.018	10	0.01
Annelida	*Prionospio dubia*			2	0.02																	16	0.05	7	0.007	10	0.03
Annelida	*Prionospio fallax*							4	0.02													2	0.01				
Annelida	*Spiophanes kroeyeri*	17	0.1	9	0.07	22	0.14	11	0.05	3	0.04	12	0.05	17	0.06	25	0.08	15	0.08	3	0.01	41	0.18	6	0.105	39	0.13
Annelida	*Exogone verugera*																					1	0.01				
Annelida	Syllidae																	2	0.01								
Annelida	*Neoamphitrite affinis*					1	2.36							4	4.32												
Annelida	*Paramphitrite tetrabranchia*	1	0.03	1	0.01	1	0.04					3	0.09			3	0.05										
Annelida	*Pista* spp.																					1	0.11			2	0.34
Annelida	*Pista cristata*	3	1.04			3	0.03							1	0.12	1	0.03			1	0.36						
Annelida	*Polycirrus* spp.	1	0.1																			1	0.02	1	0.086	1	0.09
Annelida	*Proclea graffii*	1	0.005																								
Annelida	*Streblosoma bairdi*	8	2.6	6	2.34	5	0.73	6	3.35	3	0.37	13	3.07	4	0.28	5	0.88	3	1.93	7	3.17	8	2.52	3	2.127	5	2.75
Annelida	*Terebellides stroemi*			1	0.005			1	0.04			1	0.02							1	0.01					1	0.03
Annelida	*Trichobranchus roseus*															1	0.01										
Arthropoda	*Ampelisca gibba*	1	0.01	2	0.005	1	0.005															1	0.01				
Arthropoda	*Ampelisca macrocephala*									3	0.01																
Arthropoda	*Aora gracilis*															2	0.005										
Arthropoda	*Callianassa subterranea*											1	0.005														
Arthropoda	*Diastylis boecki*							1	0.005							1	0.005										

Arthropoda	*Diastylis cornuta*																					1	0.01				
Arthropoda	*Diastyloides biplicatus*													1	0.005												
Arthropoda	*Diastyloides serratus*															1	0.005										
Arthropoda	*Gnathia oxyuraea*			2	0.005																	7	0.02				
Arthropoda	*Jassa pusilla*																					2	0.01				
Arthropoda	*Leucon nasica*																	1	0.01					1	0.005		
Arthropoda	*Leucothoe lilljeborgii*					1	0.005							2	0.005			1	0.01			1	0.01	3	0.003		
Arthropoda	*Hippomedon propinquus*																					2	0.01				
Arthropoda	Lysianassidae			1	0.02																						
Arthropoda	*Eriopisa elongata*									2	0.03																
Arthropoda	*Campylaspis costata*			1	0.005																						
Arthropoda	*Arrhis phyllonyx*													2	0.05			1	0.02			1	0.01	1	0.019		
Arthropoda	*Monoculodes packardi*																					1	0.01				
Arthropoda	*Westwoodilla caecula*					1	0.01					1	0.01					1	0.01			1	0.01			1	0.01
Arthropoda	*Harpinia antennaria*					1	0.005																				
Arthropoda	*Harpinia crenulata*	1	0.005																								
Cnidaria	Edwardsiidae																					1	0.01				
Echinodermata	*Amphiura chiajei*	3	0.01	1	0.005			2	0.13	1	0.01			4	0.17	5	0.36	4	0.49			4	0.77	3	0.398	7	0.58
Echinodermata	*Amphiura filiformis*	2	0.01	1	0.005	2	0.005																				
Echinodermata	*Brissopsis lyrifera*	4	16.08			1	12.46	1	3.98			1	4.45									2	9.34			1	6.87
Echinodermata	*Echinocardium flavescens*			2	0.005																						
Echinodermata	*Luidia sarsi*	1	0.005	1	0.005																						
Echinodermata	*Ophiocten affinis*																					2	0.01			1	0.01
Echinodermata	Spatangidae					1	0.005																				
Mollusca	Nudibranchia							1	0.01																		
Mollusca	*Chaetoderma nitidulum*					1	0.005			1	0.01											1	0.01				
Mollusca	*Cuspidaria* spp.			1	0.01																						
Mollusca	*Tropidomya abbreviata*																					1	0.01				
Mollusca	*Cylichna cylindracea*																					1	0.03	1	5E-04		
Mollusca	*Antalis entalis*																							1	0.255		
Mollusca	*Hyala vitrea*			1	0.005																			1	0.001	1	0.01
Mollusca	*Tellimya tenella*	2	0.01																			2	0.01			2	0.01
Mollusca	*Euspira montagui*																									1	0.24
Mollusca	*Ennucula tenuis*	7	0.23	3	0.21	1	0.01	2	0.13			1	0.19	1	0.005	3	0.21	1	0.09			1	0.02			1	0.08
Mollusca	*Pseudamussium peslutrae*	1	0.02					1	2.56			2	3														
Mollusca	*Hermania scabra*	1	0.01					1	0.09			1	0.08			1	0.01					2	0.01				
Mollusca	*Philine quadripartita*			2	0.01													1	0.01								
Mollusca	*Abra nitida*	2	0.01	13	0.02	1	0.01			4	0.08	1	0.01	3	0.09	1	0.005	1	0.01			2	0.01	24	0.031		
Mollusca	*Mendicula ferruginosa*																					3	0.01				
Mollusca	*Thyasira equalis*	21	0.18	36	0.15	23	0.11	10	0.13	8	0.09	13	0.3	21	0.33	18	0.26	19	0.29			33	0.43	20	0.086	29	0.26
Mollusca	*Thyasira sarsii*																	13	0.3								
Mollusca	*Mysia undata*																									1	0.01
Mollusca	*Yoldiella philippiana*	4	0.01	11	0.03	14	0.1	5	0.04	1	0.005	1	0.005	5	0.04	8	0.07	4	0.02			14	0.07	2	0.005	2	0.01
Nemertea	Nemertea			1	0.005													4	0.07	1	0.01	5	0.07	3	0.136	5	0.23
Nemertea	*Cerebratulus* spp.							1	0.02			2	0.41	2	0.46	2	0.08										
Platyhelminthes	Turbellaria													1	0.01							1	0.01	1	0.022		
Sipuncula	*Phascolion strombus*					1	0.005																				
Xenacoelomorpha	Xenoturbella																			1	0.08						

f. Ref-95

Phylum	Taxon	Ref-95:14A		Ref-95:14B		Ref-95:14C		Ref-95:14D		Ref-95:14E		Ref-95:49A		Ref-95:49B		Ref-95:49C		Ref-95:49D		Ref-95:49E	
		Abu	Bio	Abu	Bio	Abu	Bio	Abu	Bio	Abu	Bio	Abu	Bio	Abu	Bio	Abu	Bio	Abu	Bio	Abu	Bio
Annelida	*Anobothrus gracilis*					1	0.005														
Annelida	*Melinna cristata*	1	0.005	1	0.07			1	0.02	1	0.005	2	0.001	2	0.03	2	0.073				
Annelida	*Paramphinome jeffreysi*	27	0.08	13	0.03	2	0.005	5	0.01	20	0.06	45	0.195	38	0.19	17	0.069	67	0.38	17	0.09
Annelida	*Heteromastus filiformis*	9	0.04	3	0.005	17	0.04	3	0.01	11	0.03	10	0.025	24	0.05	31	0.065	40	0.16	5	0.03
Annelida	*Aphelochaeta marioni*	11	0.15	12	0.19	9	0.16	20	0.25	17	0.36	9	0.073	25	0.14	21	0.117	7	0.12	3	0.05
Annelida	*Chaetozone setosa*	30	0.2	31	0.2	20	0.17	48	0.25	28	0.23	48	0.139	67	0.16	58	0.155	76	0.39	16	0.08
Annelida	*Brada villosa*											1	0.068					2	0.01		
Annelida	*Diplocirrus glaucus*			1	0.005							1	0.019					1	0.02		
Annelida	*Glycera alba*	3	0.05					2	0.02	3	0.24					1	0.066				
Annelida	*Glycera unicornis*	1	0.18					1	0.13	1	1.46					1	0.984				
Annelida	*Goniada maculata*	2	0.05	2	0.11	2	0.05	1	0.005	1	0.005	3	0.129					2	0.1	1	0.07
Annelida	*Nereimyra punctata*							1	0.005									1	0.02		
Annelida	*Rhodine gracilior*											1	0.029	2	0.03	1	0.005			1	0.01
Annelida	*Rhodine loveni*					1	0.06	2	0.17	2	0.15					1	0.072				
Annelida	*Aglaophamus pulchra*													1	0.4						
Annelida	*Nephtys incisa*													1	0.06						
Annelida	*Ceratocephale loveni*	2	0.05	14	0.28	2	0.04	8	0.06	5	0.04	4	0.046	13	0.39	15	0.252	4	0.24	5	0.29
Annelida	*Ophelina norvegica*					1	0.06														
Annelida	*Phylo norvegica*			1	0.06							1	0.022								
Annelida	*Galathowenia oculata*							1	0.005	4	0.01	4	0.009	2	0.01	3	0.012	1	0.01		
Annelida	*Pectinaria koreni*	1	0.005	2	0.005													1	0.02		
Annelida	*Chaetoparia nilssoni*	1	0.005													2	0.008				
Annelida	*Eteone* sp.																			1	0.01
Annelida	*Phyllodoce groenlandica*											2	0.04			1	0.022				
Annelida	*Phyllodoce rosea*															2	5E-04				
Annelida	*Sige fusigera*			1	0.005			2	0.005												
Annelida	*Bylgides elegans*	1	0.03					2	0.01			3	0.024			1	0.006	2	0.11		
Annelida	Polynoidae			1	0.01					2	0.005										
Annelida	*Euchone papillosa*							1	0.005			4	0.007								
Annelida	*Lipobranchius jeffreysii*					1	0.09														
Annelida	*Polyphysia crassa*											1	0.984								
Annelida	*Scalibregma inflatum*	1	0.03	5	0.2	8	0.16	4	0.13	5	0.17	23	0.215	57	0.68	49	0.297	63	0.66	16	0.27
Annelida	*Pholoe baltica*											1	0.002								
Annelida	*Polydora* spp.									1	0.005										
Annelida	*Prionospio cirrifera*			2	0.005	4	0.02	7	0.01	2	0.005	28	0.034			4	0.005	40	0.05	2	0.01
Annelida	*Prionospio fallax*															1	0.001				
Annelida	*Spiophanes kroeyeri*	35	0.33	16	0.31	10	0.15	7	0.13	8	0.12	4	0.008	35	0.12	11	0.116	18	0.07	1	0.03
Annelida	*Pista cristata*							3	0.56							1	0.28				
Annelida	*Pistella lornensis*											1	0.186	1	0.02	3	0.514				
Annelida	*Streblosoma bairdi*							2	0.22							2	0.735			2	0.33
Annelida	*Terebellides stroemi*	2	0.03							1	0.04									1	0.38
Arthropoda	*Callianassa subterranea*			1	0.005																
Arthropoda	*Diastylis cornuta*											6	0.04					1	0.01		
Arthropoda	*Diastyloides serratus*	2	0.005	1	0.005															2	0.01
Arthropoda	*Gnathia oxyuraea*															4	0.006				
Arthropoda	*Eudorella emarginata*	6	0.005	2	0.005			2	0.005	3	0.005	6	0.006	5	0.01	1	0.001	6	0.01	2	0.01
Arthropoda	*Eudorella truncatula*											1	5E-04	3	0.005					1	0.01
Arthropoda	*Leucon nasica*	5	0.005	3	0.01	3	0.01	5	0.02	2	0.005	5	0.006	7	0.005	1	0.001	5	0.01	1	0.01

Arthropoda	*Leucothoe lilljeborgii*			1	0.005							1	0.001	1	0.005	2	0.003	1	0.01		
Arthropoda	Lysianassidae					1	0.005														
Arthropoda	*Eriopisa elongata*			1	0.005			2	0.01	1	0.005							1	0.01		
Arthropoda	*Campylaspis costata*			1	0.005																
Arthropoda	*Arrhis phyllonyx*	7	0.14	1	0.01	5	0.11	3	0.08	1	0.01					1	0.019	1	0.01	1	0.02
Arthropoda	*Bathymedon longimanus*											1	0.001								
Arthropoda	*Westwoodilla caecula*	1	0.005									1	0.001								
Arthropoda	*Philomedes brenda*											1	0.001								
Arthropoda	*Phoxocephalidae*					1	0.005														
Echinodermata	*Brissopsis lyrifera*							1	19.84									1	16.3		
Echinodermata	*Ophiocten affinis*					2	0.03											1	0.01		
Mollusca	*Tropidomya abbreviata*															1	0.037				
Mollusca	*Tellimya tenella*																	2	0.01		
Mollusca	*Ennucula tenuis*	1	0.04									1	0.006	5	0.08					1	0.04
Mollusca	*Pseudamussium peslutrae*							1	0.72												
Mollusca	*Hermania scabra*					1	0.01	3	0.16	2	0.11	2	0.038					1	0.01		
Mollusca	*Abra nitida*	2	0.04	5	0.17	3	0.05	3	0.05	9	0.19										
Mollusca	*Thyasira equalis*	3	0.08	23	0.18	1	0.03	18	0.27	23	0.46	16	0.236	25	0.27	16	0.233	14	0.3	7	0.18
Mollusca	*Yoldiella philippiana*	1	0.005			2	0.03			4	0.05	3	0.059			2	0.033			2	0.05
Nemertea	Nemertea											2	0.484			2	0.237	3	0.09		
Nemertea	*Cerebratulus* spp.			2	0.08	3	0.09			1	0.05										
Sipuncula	*Phascolion strombus*											1	0.092								

Table S-2. Species classifications. Tube-living polychaetes and molluscs with protective shells are classified as protected, marked with a *P*. Sum of abundance (abu) and the wet weight (ww) as total presence values, and the conversion factor to calculate ww to AFDW expressed in %. The bioturbation index (BPc) original values from Queirós et al (2013) are shown in brackets.
***Applies to juvenile individs adjusted to our findings, x= no value (e.g. parasite or commensal).**

								Bioturbation BPc			Bioirrigation BIPc			Bioirrigation IPc		
Aphia ID	Phylum	Family	Taxa	P	Sum abu	Sum ww	AFDW % conv. of ww	M*i*	R*i*	Ft*i*	Burrow type	Feeding type	Burrow depth (cm)	Burrow type	Feeding type	Injection pocket depth
129778	Annelida	Ampharetidae	*Ampharete finmarchica*	*P*	4	0.01	18.7%	1 (2)	2 (3)	S (UC/DC)	2	2	3	3	3	2
129818	Annelida	Ampharetidae	*Ampharete octocirrata*	*P*	1	0.00	18.7%	1	2	S	2	2	1	3	3	1
129789	Annelida	Ampharetidae	*Anobothrus gracilis*	*P*	4	0.04	18.7%	1	2 (3)	S (UC/DC)	2	2	7	3	3	3
156074	Annelida	Ampharetidae	*Eclysippe eliasoni*	*P*	21	0.07	18.7%	1	2	S	2	2	1	3	3	1
129804	Annelida	Ampharetidae	*Melinna cristata*	*P*	19	0.49	18.7%	1	2 (3)	S (UC/DC)	2	2	15	3	3	4
129813	Annelida	Ampharetidae	*Mugga wahrbergi*	*P*	5	0.00	18.7%	1 (2)	2 (3)	S (UC/DC)	2	2	1	3	3	1
129821	Annelida	Ampharetidae	*Sosane sulcata*	*P*	10	0.08	18.7%	1 (2)	2 (3)	S (UC/DC)	2	2	5	3	3	2
129837	Annelida	Amphinomidae	*Paramphinome jeffreysi*		680	2.74	13.8%	3	4	B	1	1	5	3	2	2
129884	Annelida	Capitellidae	*Heteromastus filiformis*		385	1.40	9.2%	3 (2)	3	UC	1	5	15	3	3	4
129898	Annelida	Capitellidae	*Notomastus latericeus*		6	0.39	9.2%	3 (2)	3	UC	1	5	10	3	3	3
129912	Annelida	Chaetopteridae	*Chaetopterus norvegicus*	*P*	4	2.76	14.0%	1	3	UC/DC	2	4	20	2	4	4
129924	Annelida	Chaetopteridae	*Spiochaetopterus typicus*	*P*	1	0.24	14.0%	1	2 (3)	S (UC/DC)	2	4	3	2	4	1
129240	Annelida	Cirratulidae	*Aphelochaeta* sp.		2	0.01	16.9%	3 (2)	3 (2)	DC (S)	1	2	3	3	3	1
129938	Annelida	Cirratulidae	*Aphelochaeta marioni*		334	3.88	16.9%	3 (2)	3 (2)	DC (S)	1	2	3	3	3	1
129955	Annelida	Cirratulidae	*Chaetozone setosa*		626	3.03	16.9%	3 (2)	2	S	1	2	3	3	3	1
152269	Annelida	Cirratulidae	*Tharyx killariensis*		1	0.01	16.9%	2	2	S	1	2	1	3	3	1
129984	Annelida	Cossuridae	*Cossura longocirrata*		1	0.00	12.9%	3	4 (2)	B (S)	1	5	10	3	3	3
130010	Annelida	Dorvilleidae	*Iphitime hartmanae*		1	0.08	20.5%	0	0	x	0	0	0	0	0	0
130099	Annelida	Flabelligeridae	*Brada villosa*		19	0.59	16.9%	2	2 (3)	S (UC/DC)	1	5	3	3	3	1
130100	Annelida	Flabelligeridae	*Diplocirrus glaucus*		117	0.93	16.9%	2	2 (3)	S (UC)	1	5	3	3	3	1
130116	Annelida	Glyceridae	*Glycera alba*		80	2.91	18.5%	4 (3)	4	B	3	1	10	2	2	3
130127	Annelida	Glyceridae	*Glycera unicornis*		13	6.20	18.5%	4 (3)	4	B	3	1	10	2	2	3
130136	Annelida	Goniadidae	*Glycinde nordmanni*		5	0.07	15.0%	4 (3)	4	B	1	1	3	3	2	2
130140	Annelida	Goniadidae	*Goniada maculata*		54	1.56	15.0%	4 (3)	4	B	1	1	3	3	2	2
130185	Annelida	Hesionidae	*Nereimyra punctata*		2	0.02	13.1%	3	2 (4)	S (B)	1	1	3	2	2	2
130187	Annelida	Hesionidae	*Oxydromus flexuosus*		17	0.39	13.1%	4	2	S	1	1	0	1	2	0
130197	Annelida	Hesionidae	*Podarkeopsis helgolandicus*		2	0.04	13.1%	3	2	S	1	1	3	1	2	2
152249	Annelida	Hesionidae	*Psamathe fusca*		1	0.00	13.1%	3	2 (4)	S (B)	1	1	1	1	2	1
129331	Annelida	Lumbrineridae	*Abyssoninoe* sp.		1	0.17	20.5%	3	4	B	1	1	5	3	2	2
146469	Annelida	Lumbrineridae	*Abyssoninoe hibernica*		121	3.72	20.5%	3	4	B	1	1	5	3	2	2
130244	Annelida	Lumbrineridae	*Lumbrineris gracilis*		1	0.01	20.5%	3	4	B	1	1	5	3	2	2
130261	Annelida	Lumbrineridae	*Scoletoma fragilis*		4	1.11	20.5%	3	4	B	1	1	7	3	2	4
130263	Annelida	Lumbrineridae	*Scoletoma impatiens*		1	0.04	20.5%	3	4	B	1	1	5	3	2	2
130268	Annelida	Magelonidae	*Magelona filiformis*		6	0.02	14.0%	2	2	S	1	2	3	3	3	1
130305	Annelida	Maldanidae	*Maldane sarsi*	*P*	3	0.01	15.1%	1	3	DC	2	2	10	2	3	3
923	Annelida	Maldanidae	Maldanidae	*P*	1	0.02	15.1%	3 (1)	2 (3)	S (UC)	2	5	10	2	3	3
130322	Annelida	Maldanidae	*Praxillella affinis*	*P*	39	1.26	15.1%	2 (1)	3	UC	2	5	10	2	3	3
130326	Annelida	Maldanidae	*Praxillella praetermissa*	*P*	9	0.32	15.1%	2	3	UC	2	5	10	2	3	3
130330	Annelida	Maldanidae	*Rhodine gracilior*	*P*	6	0.28	15.1%	2 (1)	3	UC	2	5	10	2	3	3
130331	Annelida	Maldanidae	*Rhodine loveni*	*P*	41	2.44	15.1%	2	3	UC	2	5	15	2	3	4
130366	Annelida	Nephtyidae	*Aglaophamus pulchra*		1	0.40	21.8%	3	4	B	1	1	5	3	2	2
130362	Annelida	Nephtyidae	*Nephtys incisa*		66	5.05	21.8%	3	4	B	1	1	5	3	2	2
130365	Annelida	Nephtyidae	*Nephtys paradoxa*		1	0.09	21.8%	3	4	B	1	1	10	3	2	3
130367	Annelida	Nereididae	*Ceratocephale loveni*		125	2.47	13.5%	3	4	B	1	1	7	3	2	3

								Bioturbation BPc			Bioirrigation BIPc			Bioirrigation IPc		
Aphia ID	Phylum	Family	Taxa	P	Sum abu	Sum ww	AFDW % conv. of ww	M*i*	R*i*	Ft*i*	Burrow type	Feeding type	Burrow depth (cm)	Burrow type	Feeding type	Injection pocket depth
129856	Annelida	Oenonidae	*Drilonereis filum*		1	0.50	20.5%	3	4	B	1	1	10	3	2	3
129414	Annelida	Opheliidae	*Ophelina* sp.		1	0.00	15.8%	3	4	B	1	5	5	3	3	2
130508	Annelida	Opheliidae	*Ophelina norvegica*		3	0.11	15.8%	3	4	B	1	5	5	3	3	2
130531	Annelida	Orbiniidae	*Phylo norvegica*		3	0.45	9.3%	3	4	B	1	5	10	3	3	3
146950	Annelida	Oweniidae	*Galathowenia oculata*	*P*	34	0.13	21.4%	1	2	S	2	2	1	2	3	1
130544	Annelida	Oweniidae	*Owenia fusiformis*	*P*	1	0.01	21.4%	1	2	S	2	2	1	2	3	1
130578	Annelida	Paraonidae	*Levinsenia gracilis*		9	0.01	12.9%	3	2	S	1	5	3	3	3	2
130590	Annelida	Pectinariidae	*Pectinaria auricoma*	*P*	9	0.23	6.2%	2 (1)	3	UC	2	5	5	3	3	2
130592	Annelida	Pectinariidae	*Pectinaria belgica*	*P*	29	14.41	6.2%	2	3	UC	2	5	7	3	3	2
130595	Annelida	Pectinariidae	*Pectinaria koreni*	*P*	126	3.58	6.2%	2	3	UC	2	5	5	3	3	2
130610	Annelida	Phyllodocidae	*Chaetoparia nilssoni*		6	0.03	7.3%	3	4	B	1	1	1	3	2	1
129443	Annelida	Phyllodocidae	*Eteone* sp.		2	0.01	7.3%	3	4	B	1	1	1	3	2	1
130616	Annelida	Phyllodocidae	*Eteone longa*		1	0.00	7.3%	3	4	B	1	1	1	3	2	1
130641	Annelida	Phyllodocidae	*Eumida bahusiensis*		1	0.00	7.3%	3	4	B	1	1	1	3	2	1
130656	Annelida	Phyllodocidae	*Nereiphylla lutea*		2	0.01	7.3%	3	4	B	1	1	1	3	2	1
334506	Annelida	Phyllodocidae	*Phyllodoce groenlandica*		10	0.10	7.3%	3	4	B	1	1	3	3	2	2
334514	Annelida	Phyllodocidae	*Phyllodoce rosea*		5	0.02	7.3%	3	4	B	1	1	3	3	2	2
931	Annelida	Phyllodocidae	Phyllodocidae		1	0.00	7.3%	3	4	B	1	1	1	3	2	1
130690	Annelida	Phyllodocidae	*Sige fusigera*		4	0.01	7.3%	3	4	B	1	1	1	3	2	1
130696	Annelida	Pilargidae	*Glyphohesione klatti*		1	0.01	13.1%	3	4	B	1	1	1	3	2	1
130732	Annelida	Polynoidae	*Bylgides elegans*		23	0.27	12.4%	3	2	S	1	1	1	3	2	1
130745	Annelida	Polynoidae	*Eunoe nodosa*		1	0.00	12.4%	3	2 (4)	S (B)	1	1	1	3	2	1
130748	Annelida	Polynoidae	*Gattyana amondseni*		2	0.01	12.4%	3	2 (4)	S (B)	1	1	1	3	2	1
130749	Annelida	Polynoidae	*Gattyana cirrhosa*		2	0.00	12.4%	3	2 (4)	S (B)	1	1	3	3	2	2
129491	Annelida	Polynoidae	*Harmothoe* sp.		2	0.01	12.4%	3	2 (4)	S (B)	1	1	1	3	2	1
130758	Annelida	Polynoidae	*Harmothoe borealis*		3	0.01	12.4%	3	2	S	1	1	1	3	2	1
939	Annelida	Polynoidae	Polynoidae		12	0.05	12.4%	3	2 (4)	S (B)	1	1	1	3	2	1
130906	Annelida	Sabellidae	*Euchone papillosa*	*P*	22	0.12	21.4%	1	2	S	2	4	1	3	1	1
130920	Annelida	Sabellidae	*Jasmineira caudata*	*P*	3	0.00	21.4%	1	2	S	2	4	1	3	1	1
333843	Annelida	Scalibregmatidae	*Lipobranchius jeffreysii*		35	13.21	12.9%	4	4	B	3	5	10	2	3	4
130977	Annelida	Scalibregmatidae	*Polyphysia crassa*		6	3.23	12.9%	4	4	B	3	5	10	2	3	4
130980	Annelida	Scalibregmatidae	*Scalibregma inflatum*		621	6.48	12.9%	4	4	B	3	5	10	2	3	4
130599	Annelida	Sigalionidae	*Pholoe baltica*		20	0.08	13.1%	3 (2)	2	S	1	1	1	3	2	1
130604	Annelida	Sigalionidae	*Pholoe pallida*		5	0.03	13.1%	3 (2)	2	S	1	1	1	3	2	1
131127	Annelida	Spionidae	*Laonice bahusiensis*		2	0.01	17.8%	2 (1)	2 (3)	S (UC/DC)	1	2	5	3	3	2
129619	Annelida	Spionidae	*Polydora* sp.	*P*	28	0.05	17.8%	1	2 (3)	S (UC/DC)	2	2	1	3	3	1
131153	Annelida	Spionidae	*Prionospio cirrifera*	*P*	283	0.50	17.8%	1 (2)	2 (3)	S (UC/DC)	2	2	1	3	3	1
131155	Annelida	Spionidae	*Prionospio dubia*	*P*	38	0.15	17.8%	1 (2)	2 (3)	S (UC/DC)	2	2	1	3	3	1
131157	Annelida	Spionidae	*Prionospio fallax*	*P*	15	0.05	17.8%	1 (2)	2 (3)	S (UC/DC)	2	2	1	3	3	1
157561	Annelida	Spionidae	*Scolelepis tridentata*		3	0.01	17.8%	2	2	S	1	2	3	3	3	2
157576	Annelida	Spionidae	*Spiophanes kroeyeri*	*P*	520	4.25	17.8%	1	2	S	2	2	5	3	3	2
131307	Annelida	Syllidae	*Exogone verugera*		1	0.00	10.6%	3	4	B	1	1	1	1	2	1
948	Annelida	Syllidae	Syllidae		2	0.00	10.6%	3	2	S	1	1	1	1	2	1
131502	Annelida	Terebellidae	*Neoamphitrite affinis*	*P*	7	6.76	12.5%	1	3	DC	2	2	10	3	3	3
131505	Annelida	Terebellidae	*Neoamphitrite grayi*	*P*	1	0.61	12.5%	1	3	UC/DC	2	2	10	3	3	3
131511	Annelida	Terebellidae	*Paramphitrite tetrabranchia*	*P*	9	0.22	12.5%	1	3	UC/DC	2	2	3	3	3	2
129708	Annelida	Terebellidae	*Pista* sp.	*P*	3	0.45	12.5%	1	3	UC/DC	2	2	3	3	3	2

Aphia ID	Phylum	Family	Taxa	P	Sum abu	Sum ww	AFDW % conv. of ww	Bioturbation BPc			Bioirrigation BIPc			Bioirrigation IPc		
								M*i*	R*i*	Ft*i*	Burrow type	Feeding type	Burrow depth (cm)	Burrow type	Feeding type	Injection pocket depth
131516	Annelida	Terebellidae	*Pista cristata*	*P*	17	2.80	12.5%	1	3	DC	2	2	3	3	3	2
131522	Annelida	Terebellidae	*Pistella lornensis*	*P*	5	0.72	12.5%	1	3	DC	2	2	3	3	3	2
129710	Annelida	Terebellidae	*Polycirrus* sp.		17	0.55	12.5%	2 (1)	3	DC	3	2	1	3	3	1
157545	Annelida	Terebellidae	*Proclea graffii*		1	0.00	12.5%	2	3	UC/DC	3	2	1	3	3	1
131538	Annelida	Terebellidae	*Streblosoma bairdi*	*P*	87	27.58	12.5%	1	3	DC	2	2	7	3	3	3
183466	Annelida	Terebellidae	*Terebellides stroemi*	*P*	48	0.95	13.5%	2 (1)	2 (3)	S (DC)	1	2	3	3	3	2
131575	Annelida	Terebellidae	*Trichobranchus roseus*		14	0.24	13.5%	2 (1)	2 (3)	S (DC)	1	5	3	3	3	2
1133	Arthropoda		Tanaidacea		3	0.01	16.7%	2	2	S	2	1	1	3	2	1
101898	Arthropoda	Ampeliscidae	*Ampelisca gibba*		5	0.02	41.3%	1	2	S	1	2	1	1	3	1
101908	Arthropoda	Ampeliscidae	*Ampelisca macrocephala*		3	0.01	41.3%	1	2	S	2	2	1	1	3	1
101930	Arthropoda	Ampeliscidae	*Ampelisca tenuicornis*		2	0.01	41.3%	1	2	S	2	2	1	1	3	1
102012	Arthropoda	Aoridae	*Aora gracilis*		2	0.00	17.6%	3	2 (1)	S (S)	3	2	1	2	3	1
107729	Arthropoda	Callianassidae	*Callianassa subterranea*		32	1.28	21.8%	4	4	B	3	2	15	2	3	4
110471	Arthropoda	Diastylidae	*Diastylis boecki*		4	0.03	16.0%	3	2	S	1	2	1	3	3	1
110474	Arthropoda	Diastylidae	*Diastylis cornuta*		11	0.07	16.0%	3	2	S	1	2	1	3	3	1
110481	Arthropoda	Diastylidae	*Diastylis laevis*		1	0.02	16.0%	3	2	S	1	2	1	3	3	1
110494	Arthropoda	Diastylidae	*Diastyloides biplicatus*		1	0.00	16.0%	2	2	S	1	2	1	3	3	1
110497	Arthropoda	Diastylidae	*Diastyloides serratus*		13	0.03	16.0%	3	2	S	1	2	1	3	3	1
110503	Arthropoda	Diastylidae	*Leptostylis longimana*		1	0.00	16.0%	3	2	S	1	2	1	3	3	1
118995	Arthropoda	Gnathiidae	*Gnathia oxyuraea*		14	0.04	16.7%	1 (3)	2	S	3	0	1	2	0	1
102437	Arthropoda	Ischyroceridae	*Jassa pusilla*		2	0.00	18.9%	1	2	S	0	4	0	1	1	0
110524	Arthropoda	Leuconidae	*Eudorella emarginata*		61	0.14	16.0%	3	2	S	1	5	5	3	3	3
110535	Arthropoda	Leuconidae	*Eudorella truncatula*		8	0.02	16.0%	3	2	S	1	5	3	3	3	2
110618	Arthropoda	Leuconidae	*Leucon nasica*		85	0.24	16.0%	3	2	S	1	2	1	3	3	1
102462	Arthropoda	Leucothoidae	*Leucothoe lilljeborgii*		15	0.05	18.9%	3	2	S	1	2	1	3	3	1
102579	Arthropoda	Lysianassidae	*Hippomedon propinquus*		4	0.02	20.8%	3	2	S	1	1	3	3	2	2
101395	Arthropoda	Lysianassidae	Lysianassidae		3	0.03	20.8%	3	2	S	1	1	1	3	2	1
102807	Arthropoda	Melitidae	*Eriopisa elongata*		18	0.09	18.9%	4 (2)	4 (2)	B (S)	3	5	7	3	3	3
110542	Arthropoda	Nannastacidae	*Campylaspis costata*		3	0.01	16.0%	3	2	S	1	1	1	3	2	1
102870	Arthropoda	Oedicerotidae	*Arrhis phyllonyx*		44	0.87	18.9%	3	2	S	1	2	1	3	3	1
102873	Arthropoda	Oedicerotidae	*Bathymedon longimanus*		1	0.00	18.9%	3	2	S	1	2	1	3	3	1
102882	Arthropoda	Oedicerotidae	*Monoculodes carinatus*		1	0.00	18.9%	3	2	S	1	2	1	3	3	1
102892	Arthropoda	Oedicerotidae	*Monoculodes packardi*		1	0.00	18.9%	3	2	S	1	2	1	3	3	1
101400	Arthropoda	Oedicerotidae	Oedicerotidae		1	0.00	18.9%	3	2	S	1	2	1	3	3	1
102932	Arthropoda	Oedicerotidae	*Westwoodilla caecula*		14	0.09	18.9%	3	2	S	1	2	1	3	3	1
127718	Arthropoda	Philomedidae	*Philomedes brenda*		18	0.04	15.3%	3	2	S	0	2	0	1	3	0
102960	Arthropoda	Phoxocephalidae	*Harpinia antennaria*		1	0.00	11.5%	3	2	S	1	5	3	3	3	1
102963	Arthropoda	Phoxocephalidae	*Harpinia crenulata*		1	0.00	11.5%	3	2	S	1	5	3	3	3	1
101403	Arthropoda	Phoxocephalidae	Phoxocephalidae		1	0.00	11.5%	3	2	S	1	5	3	3	3	1
100665	Cnidaria	Edwardsiidae	Edwardsiidae		15	0.16	11.6%	2	2	S	3	1	3	3	2	1
125073	Echinodermata	Amphiuridae	*Amphiura chiajei*		88	9.57	12.9%	3	4	B	1	2	5	2	3	2
125080	Echinodermata	Amphiuridae	*Amphiura filiformis*		863	60.32	15.4%	3	4	B	1	4	5	2	3	2
123803	Echinodermata	Asteriidae	*Marthasterias glacialis*		1	0.01	17.1%	4	1	E	1	1	0	1	2	0
124373	Echinodermata	Brissidae	*Brissopsis lyrifera*		48	380.42	3.5%	3	4	B	1	5	10	3	3	3
124635	Echinodermata	Cucumariidae	*Leptopentacta elongata*		5	1.29	12.6%	2 (3)	2	S	1	2	3	3	3	1
124392	Echinodermata	Loveniidae	*Echinocardium cordatum*		15	56.62	3.5%	3	4	B	1	5	7	3	3	3
124394	Echinodermata	Loveniidae	*Echinocardium flavescens*		32	43.94	3.5%	3	4	B	1	5	7	3	3	3

								Bioturbation BPc			Bioirrigation BIPc			Bioirrigation IPc		
Aphia ID	Phylum	Family	Taxa	P	Sum abu	Sum ww	AFDW % conv. of ww	M*i*	R*i*	Ft*i*	Burrow type	Feeding type	Burrow depth (cm)	Burrow type	Feeding type	Injection pocket depth
178639	Echinodermata	Luidiidae	*Luidia sarsi*		7	0.04	12.2%	3	2	S	1	1	1	3	2	1
124850	Echinodermata	Ophiuridae	*Ophiocten affinis*		7	0.13	12.3%	3 (2)	2	S	1	1	0	1	2	0
124913	Echinodermata	Ophiuridae	*Ophiura albida*		1	0.00	12.3%	3 (2)	2	S	1	1	0	1	2	0
123177	Echinodermata	Spatangidae	Spatangidae		1	0.00	3.5%	3	2* (4)	S* (B)	1	5	1	3	3	1
816800	Echinodermata	Synallactidae	*Mesothuria intestinalis*		1	34.70	4.0%	2	2	S	1	2	0	1	3	0
124455	Echinodermata	Synaptidae	*Labidoplax buskii*		2	0.06	4.0%	2 (3)	2	S	1	2	1	3	3	1
1762	Mollusca		Nudibranchia		2	0.03	17.2%	2	2	S	1	1	0	1	2	0
139010	Mollusca	Cardiidae	*Parvicardium minimum*	*P*	2	0.06	5.8%	2	2	S	1	3	1	1	1	1
181343	Mollusca	Cardiidae	*Parvicardium pinnulatum*	*P*	3	0.14	5.8%	2	2	S	1	3	1	1	1	1
139106	Mollusca	Chaetodermatidae	*Chaetoderma nitidulum*		10	0.40	13.8%	3 (2)	2	S	1	1	3	3	2	2
139410	Mollusca	Corbulidae	*Corbula gibba*	*P*	109	8.40	5.8%	2	2	S	1	3	3	1	1	2
137858	Mollusca	Cuspidariidae	*Cuspidaria* sp.	*P*	1	0.01	5.8%	2	4	B	1	1	3	3	2	2
139450	Mollusca	Cuspidariidae	*Cuspidaria obesa*	*P*	1	0.08	5.8%	2	4 (3)	B (UC/DC)	1	1	3	3	2	2
139470	Mollusca	Cuspidariidae	*Tropidomya abbreviata*	*P*	3	0.09	5.8%	2	4	B	1	1	3	3	2	2
139476	Mollusca	Cylichnidae	*Cylichna cylindracea*	*P*	31	0.41	13.8%	3	2	S	1	1	1	3	2	1
150534	Mollusca	Dentaliidae	*Antalis entalis*	*P*	4	2.81	7.5%	2	3	UC	1	1	3	3	2	2
140129	Mollusca	Iravadiidae	*Hyala vitrea*	*P*	58	0.19	7.5%	3	4 (2)	B (S)	1	5	10	3	3	3
140380	Mollusca	Lasaeidae	*Kurtiella bidentata*	*P*	4	0.01	5.8%	2	4	B	1	4	5	3	3	2
140371	Mollusca	Lasaeidae	*Tellimya ferruginosa*	*P*	6	0.02	5.8%	2	4	B	1	5	7	3	3	3
152397	Mollusca	Lasaeidae	*Tellimya tenella*	*P*	16	0.05	5.8%	2	4 (2)	B (S)	1	5	10	3	3	3
140287	Mollusca	Lucinidae	*Myrtea spinifera*	*P*	1	0.03	5.8%	2	4 (2)	B (S)	1	4	5	1	1	2
150638	Mollusca	Naticidae	*Euspira montagui*	*P*	1	0.24	7.5%	3	2	S	1	1	3	3	2	2
428773	Mollusca	Naticidae	*Euspira nitida*	*P*	5	0.40	7.5%	3	2	S	1	1	3	3	2	2
140584	Mollusca	Nuculidae	*Ennucula tenuis*	*P*	33	1.47	5.8%	3	2	S	1	5	3	3	3	2
140589	Mollusca	Nuculidae	*Nucula nitidosa*	*P*	93	3.52	5.8%	3	2	S	1	5	3	3	3	2
140717	Mollusca	Pectinidae	*Pseudamussium peslutrae*	*P*	5	6.30	5.8%	3	2	S	0	4	0	1	1	0
140737	Mollusca	Pharidae	*Phaxas pellucidus*	*P*	2	0.03	5.8%	2	4 (2)	B (S)	1	3	3	1	1	2
140761	Mollusca	Philinidae	*Hermania scabra*		73	1.37	13.8%	3	2	S	1	1	1	3	2	1
140744	Mollusca	Philinidae	*Philine quadripartita*		7	0.05	13.8%	3	2	S	1	1	1	3	2	1
141433	Mollusca	Semelidae	*Abra alba*	*P*	1	0.00	5.8%	2	4 (2)	B (S)	1	2	3	1	3	2
141435	Mollusca	Semelidae	*Abra nitida*	*P*	163	5.51	5.8%	2	4 (2)	B (S)	1	2	3	1	3	2
152905	Mollusca	Thyasiridae	*Mendicula ferruginosa*	*P*	3	0.01	5.8%	2	4 (2)	B (S)	1	4	3	1	1	2
141659	Mollusca	Thyasiridae	*Thyasira equalis*	*P*	634	9.66	5.8%	2	4 (3)	B (DC)	1	4	5	1	1	3
141662	Mollusca	Thyasiridae	*Thyasira flexuosa*	*P*	43	0.69	5.8%	2	4 (3)	B (DC)	1	4	5	1	1	3
141672	Mollusca	Thyasiridae	*Thyasira sarsii*	*P*	21	0.47	5.8%	2	4 (3)	B (DC)	1	4	7	1	1	3
140728	Mollusca	Veneridae	*Mysia undata*	*P*	2	0.12	5.8%	2	4 (2)	B (S)	1	3	3	1	1	2
142005	Mollusca	Yoldiidae	*Yoldiella philippiana*	*P*	140	1.47	5.8%	3	2	S	1	2	1	3	3	1
152391	Nemertea		Nemertea		39	3.79	20.0%	3	4	B	1	1	3	3	2	2
122348	Nemertea	Heteronemertea, genera incertae sedis	*Cerebratulus* spp.		21	19.44	19.8%	3	4	B	1	1	3	3	2	2
794	Platyhelminthes		Turbellaria		5	0.34	25.2%	3 (2)	2	S	1	1	0	1	2	0
136050	Sipuncula	Golfingiidae	*Golfingia vulgaris*		3	0.82	13.9%	2	4	B	1	2	5	3	3	3
136063	Sipuncula	Golfingiidae	*Thysanocardia procera*		22	1.65	13.9%	2 (3)	4	B	1	4	3	3	1	2
175043	Sipuncula	Phascolionidae	*Phascolion strombus*		14	0.11	13.9%	1	2	S	1	2	1	1	3	1
138665	Xenacoelomorpha	Xenoturbellidae	*Xenoturbella* sp.		1	0.08	16.0%	3	4	B	1	1	3	3	2	2

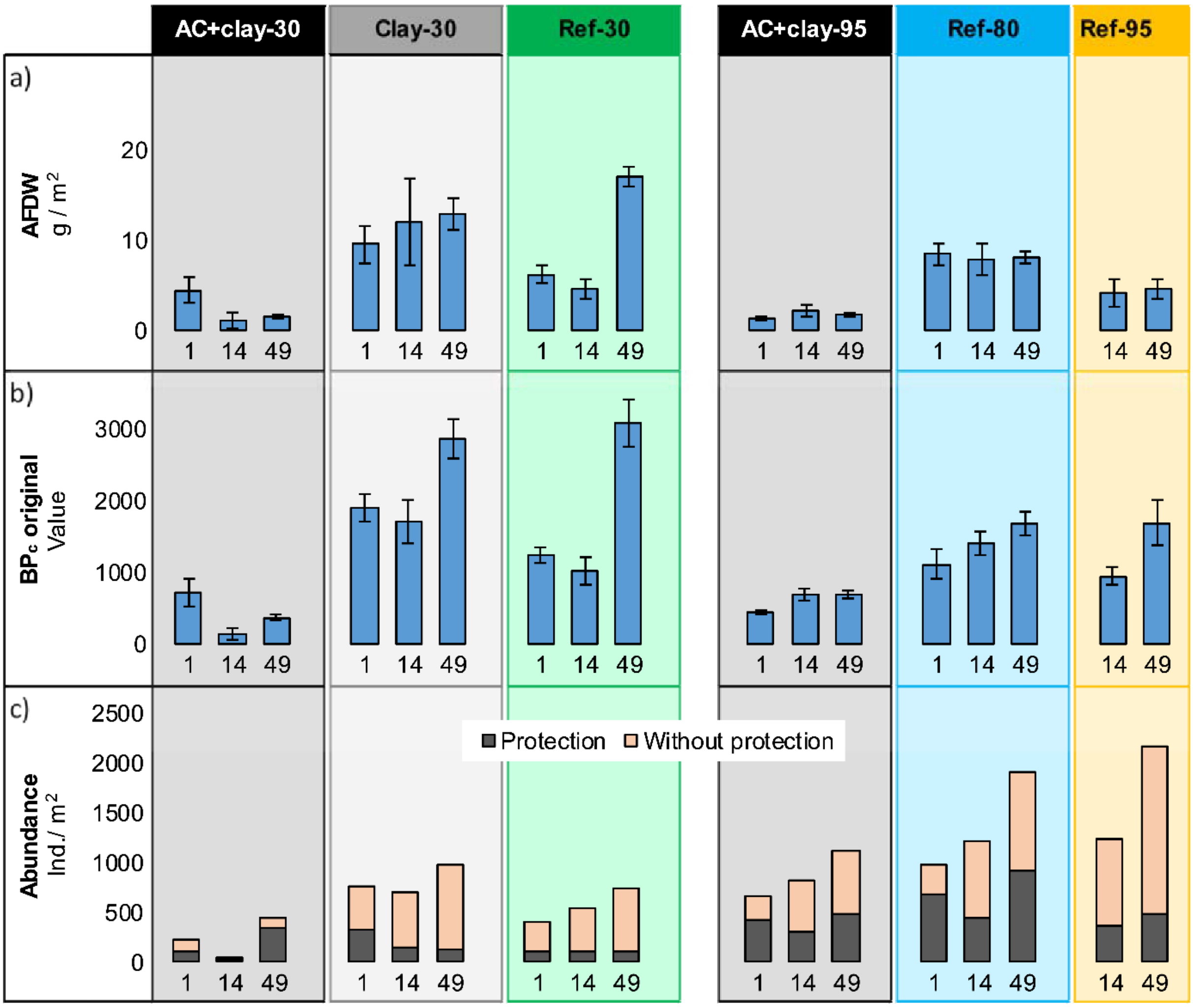

Figure S-1. Additional biometrics. a) Biomass as AFDW, b) BPc with original values from Queirós et al (2013), c) Abundance per m^2, showing species with protection (molluscs and polychaetes with tubes) and without protection (see species classifications Table S-2).

Table S-3. PERMANOVA main test. Results from the overall PERMANOVA main test analyses of univariate community metrics using planned comparison between treatments. Significant *p*-values are shown in bold numbers, α = 0.05. *Df* degrees of freedom, *num* numerator, *den* denominator, *PsF* pseudo-F value, *P(perm) p*-value by permutation.

		Df (Num,	No. of species		Abundance		Biomass		BP_c		BIP_c		IP_c	
Depth	Factor	Den)	*PsF*	P(perm)	*PsF*	P(perm)	*PsF*	P(perm)	*PsF*	P(perm)	*PsF*	P(perm)	*PsF*	P(perm)
30 m	Treatment (n=3)	2, 30	*35.69*	**0.0001**	*70.68*	**0.0001**	*13.03*	**0.0001**	*60.28*	**0.0001**	*41.15*	**0.0001**	*28.99*	**0.0001**
	AC+clay-30 vs Ref-30	1, 20	*47.87*	**0.0001**	*52.20*	**0.0001**	*17.28*	**0.0007**	*73.55*	**0.0001**	*43.66*	**0.0001**	*42.47*	**0.0001**
	AC+clay-30 vs Clay-30	1, 20	*70.62*	**0.0001**	*140.67*	**0.0001**	*21.54*	**0.0003**	*111.20*	**0.0001**	*76.55*	**0.0001**	*44.51*	**0.0001**
	Clay-30 vs Ref-30	1, 20	*3.01*	0.0986	*18.33*	**0.0008**	*0.43*	0.5189	*4.41*	0.0501	*0.79*	0.3828	*0.60*	0.4483
	Month (n=3)	2, 4	*2.62*	0.2082	*5.30*	0.0803	*1.70*	0.2822	*6.58*	0.0689	*1.94*	0.2520	*2.92*	0.1696
	Treatment*Month	4, 30	*3.99*	**0.0100**	*4.71*	**0.0045**	*2.86*	**0.0433**	*3.95*	**0.0108**	*2.55*	0.0578	*3.76*	**0.0160**
	AC+clay-30 vs Ref-30*Month	2, 20	*10.04*	**0.0005**	*6.98*	**0.0049**	*4.27*	**0.0289**	*7.73*	**0.0047**	*3.98*	**0.0351**	*8.00*	**0.0029**
	AC+clay-30 vs Clay-30*Month	2, 20	*2.96*	0.0715	*5.64*	**0.0098**	*2.15*	0.1397	*2.66*	0.0933	*1.48*	0.2509	*1.68*	0.2161
	Clay-30 vs Ref-30*Month	2, 20	*1.04*	0.3706	*0.94*	0.4016	*2.26*	0.1300	*1.96*	0.1628	*1.56*	0.2338	*2.23*	0.1346
80-95 m	Treatment (n=3)	2, 28	*17.94*	**0.0001**	*7.32*	**0.0023**	*19.52*	**0.0001**	*24.22*	**0.0001**	*57.56*	**0.0001**	*57.40*	**0.0001**
	AC+clay-95 vs Ref-80	1, 20	*33.77*	**0.0001**	*12.17*	**0.0023**	*51.51*	**0.0001**	*68.91*	**0.0001**	*116.34*	**0.0001**	*132.13*	**0.0001**
	AC+clay-95 vs Ref-95	1, 18	*3.10*	0.0950	*11.31*	**0.0029**	*4.55*	**0.0477**	*16.79*	**0.0006**	*53.02*	**0.0001**	*28.05*	**0.0001**
	Ref-80 vs Ref-95	1, 18	*11.07*	**0.0040**	*0.18*	0.6801	*6.08*	**0.0255**	*1.55*	0.2302	*8.06*	**0.0086**	*14.52*	**0.0018**
	Month (n=3)	2, 4	*3.13*	0.1904	*32.03*	**0.0192**	*0.03*	0.9638	*5.58*	0.1016	*1.51*	0.3484	*0.87*	0.4952
	Month*Treatment	3, 28	*0.40*	0.7635	*0.21*	0.8959	*0.99*	0.4173	*1.22*	0.3306	*3.53*	**0.0281**	*0.71*	0.5587
	AC+clay-95 vs Ref-80*Month	2, 20	*0.11*	0.8974	*0.18*	0.8397	*1.44*	0.2575	*0.59*	0.5610	*3.11*	0.0651	*0.65*	0.5292
	AC+clay-95 vs Ref-95*Month	1, 18	*0.54*	0.4700	*0.45*	0.5139	*1.18*	0.2914	*2.98*	0.1019	*3.84*	0.0669	*1.74*	0.1972
	Ref-80 vs Ref-95*Month	1, 18	*0.96*	0.3388	*0.08*	0.7806	*0.09*	0.7662	*0.97*	0.3407	*3.27*	0.0847	*0.12*	0.7352

Table S-4. Most important species in each field after 1, 14 and 49 months, for a) Individs (Ind.) average per grab, b) Bioturbation BPc, c) Bioirrigation BIPc, d) Bioirrigation IPc. Percentage (%) is proportion of the total value.

a)		1 month				14 months				49 months			
		Phylum	*Taxa*	Ind.	%	Phylum	*Taxa*	Ind.	%	Phylum	*Taxa*	Ind.	%
30 m	AC+clay-30	Mollusca	*Hyala vitrea*	3.0	12.0%	Annelida	*Abyssoninoe hibernica*	1.0	15.6%	Annelida	*Pectinaria koreni*	23.6	45.6%
		Mollusca	*Cylichna cylindracea*	2.7	10.7%	Annelida	*Nephtys incisa*	0.8	12.5%	Mollusca	*Nucula nitidosa*	10.4	20.1%
		Annelida	*Nephtys incisa*	2.3	9.3%	Mollusca	*Corbula gibba*	0.8	12.5%	Annelida	*Nephtys incisa*	2.8	5.4%
		Mollusca	*Corbula gibba*	1.7	6.7%	Mollusca	*Thyasira sarsii*	0.6	9.4%	Mollusca	*Thyasira flexuosa*	2.8	5.4%
		Mollusca	*Tellimya ferruginosa*	1.7	6.7%	Annelida	*Scalibregma inflatum*	0.4	6.3%	Mollusca	*Abra nitida*	2.6	5.0%
		Mollusca	*Tellimya tenella*	1.7	6.7%	Mollusca	*Hermania scabra*	0.4	6.3%	Mollusca	*Hermania scabra*	1.6	3.1%
		Annelida	*Scalibregma inflatum*	1.3	5.3%	Annelida	*Spiophanes kroeyeri*	0.4	6.3%	Annelida	*Heteromastus filiformis*	1.6	3.1%
		Annelida	*Trichobranchus roseus*	1.3	5.3%	*10 species with the same abundance, among:*				Annelida	*Magelona filiformis*	1.0	1.9%
		Echinodermat	*Echinocardium cordatum*	1.3	5.3%	Echinodermata	*Amphiura filiformis*	0.2	3.1%	Mollusca	*Corbula gibba*	0.8	1.5%
										Annelida	*Diplocirrus glaucus*	0.8	1.5%
			Sum	17.0	68.0%		Sum	4.6	71.9%		Sum	48.0	92.7%
			Tot	25.0	100%		Tot	6.4	100%		Tot	51.8	100%
	Clay-30	Echinodermat	*Amphiura filiformis*	29.3	33.1%	Annelida	*Scalibregma inflatum*	27.4	33.7%	Echinodermat	*Amphiura filiformis*	67.6	59.2%
		Mollusca	*Corbula gibba*	10.0	11.3%	Echinodermata	*Amphiura filiformis*	20.6	25.3%	Echinodermat	*Amphiura chiajei*	4.0	3.5%
		Mollusca	*Hyala vitrea*	8.3	9.4%	Mollusca	*Corbula gibba*	5.6	6.9%	Annelida	*Pectinaria belgica*	3.4	3.0%
		Mollusca	*Nucula nitidosa*	7.7	8.6%	Annelida	*Polydora*	2.2	2.7%	Mollusca	*Corbula gibba*	3.2	2.8%
		Annelida	*Abyssoninoe hibernica*	4.0	4.5%	Mollusca	*Thyasira flexuosa*	2.0	2.5%	Mollusca	*Hermania scabra*	3.2	2.8%
		Mollusca	*Thyasira flexuosa*	3.0	3.4%	Annelida	*Nephtys incisa*	2.0	2.5%	Annelida	*Abyssoninoe hibernica*	2.6	2.3%
		Annelida	*Diplocirrus glaucus*	2.3	2.6%	Echinodermata	*Amphiura chiajei*	1.6	2.0%	Annelida	*Diplocirrus glaucus*	2.4	2.1%
		Echinodermat	*Amphiura chiajei*	2.3	2.6%	Mollusca	*Nucula nitidosa*	1.4	1.7%	Annelida	*Scalibregma inflatum*	2.0	1.8%
		Sipuncula	*Thysanocardia procera*	1.7	1.9%	Annelida	*Diplocirrus glaucus*	1.4	1.7%	Annelida	*Chaetozone setosa*	1.8	1.6%
			Sum	68.7	77.4%		Sum	64.2	78.9%		Sum	90.2	79.0%
			Tot	88.7	100%		Tot	81.4	100%		Tot	114.2	100%
	Ref-30	Echinodermat	*Amphiura filiformis*	21.7	45.1%	Annelida	*Scalibregma inflatum*	26.4	42.3%	Echinodermat	*Amphiura filiformis*	43.4	50.3%
		Mollusca	*Corbula gibba*	6.0	12.5%	Echinodermata	*Amphiura filiformis*	8.2	13.1%	Echinodermat	*Echinocardium flavescens*	5.0	5.8%
		Mollusca	*Cylichna cylindracea*	2.3	4.9%	Annelida	*Diplocirrus glaucus*	3.0	4.8%	Annelida	*Abyssoninoe hibernica*	4.0	4.6%
		Annelida	*Abyssoninoe hibernica*	1.7	3.5%	Annelida	*Terebellides stroemi*	3.0	4.8%	Annelida	*Praxillella affinis*	3.8	4.4%
		Mollusca	*Hyala vitrea*	1.3	2.8%	Annelida	*Spiophanes kroeyeri*	2.2	3.5%	Annelida	*Diplocirrus glaucus*	2.8	3.2%
		Annelida	*Spiophanes kroeyeri*	1.3	2.8%	Arthropoda	*Callianassa subterrane*	2.0	3.2%	Annelida	*Terebellides stroemi*	2.4	2.8%
		Echinodermat	*Brissopsis lyrifera*	1.3	2.8%	Annelida	*Abyssoninoe hibernica*	1.6	2.6%	Echinodermat	*Brissopsis lyrifera*	2.4	2.8%
		Arthropoda	*Callianassa subterranea*	1.3	2.8%	Annelida	*Oxydromus flexuosus*	1.6	2.6%	Echinodermat	*Amphiura chiajei*	2.2	2.6%
						Annelida	*Nephtys incisa*	1.2	1.9%	Annelida	*Goniada maculata*	1.8	2.1%
			Sum	37.0	77.1%		Sum	49.2	78.8%		Sum	67.8	78.7%
			Tot	48.0	100%		Tot	62.4	100%		Tot	86.2	100%
80-95 m	AC+clay-95	Mollusca	*Thyasira equalis*	22.7	29.3%	Annelida	*Paramphinome jeffreysi*	28.8	30.4%	Annelida	*Paramphinome jeffreysi*	22.4	17.4%
		Mollusca	*Yoldiella philippiana*	9.0	11.6%	Mollusca	*Thyasira equalis*	12.0	12.7%	Mollusca	*Thyasira equalis*	21.8	16.9%
		Annelida	*Spiophanes kroeyeri*	8.0	10.3%	Mollusca	*Abra nitida*	10.0	10.5%	Annelida	*Prionospio cirrifera*	17.0	13.2%
		Annelida	*Chaetozone setosa*	4.0	5.2%	Annelida	*Spiophanes kroeyeri*	9.0	9.5%	Annelida	*Aphelochaeta marioni*	12.2	9.5%
		Sipuncula	*Phascolion strombus*	4.0	5.2%	Annelida	*Aphelochaeta marioni*	4.0	4.2%	Annelida	*Spiophanes kroeyeri*	10.6	8.2%
		Annelida	*Paramphinome jeffreysi*	3.3	4.3%	Annelida	*Chaetozone setosa*	3.2	3.4%	Arthropoda	*Leucon nasica*	7.6	5.9%
		Annelida	*Ceratocephale loveni*	2.7	3.4%	Annelida	*Heteromastus filiformis*	3.2	3.4%	Annelida	*Heteromastus filiformis*	7.0	5.4%
		Annelida	*Aphelochaeta marioni*	2.3	3.0%	Annelida	*Ceratocephale loveni*	2.6	2.7%	Arthropoda	*Eudorella emarginata*	3.8	3.0%
		Arthropoda	*Diastyloides serratus*	2.0	2.6%	Arthropoda	*Arrhis phyllonyx*	2.6	2.7%	Mollusca	*Yoldiella philippiana*	3.4	2.6%
		Arthropoda	*Philomedes brenda*	2.0	2.6%					Annelida	*Chaetozone setosa*	2.2	1.7%
				60.0	77.6%		Sum	75.4	79.5%		Sum	108.0	83.9%
				77.3	100%		Tot	94.8	100%		Tot	128.8	100%
	Ref-80	Mollusca	*Thyasira equalis*	26.7	23.3%	Annelida	*Paramphinome jeffreysi*	25.2	17.9%	Annelida	*Heteromastus filiformis*	22.4	10.1%
		Annelida	*Spiophanes kroeyeri*	16.0	14.0%	Mollusca	*Thyasira equalis*	14.0	10.0%	Annelida	*Prionospio cirrifera*	21.2	9.5%
		Mollusca	*Yoldiella philippiana*	9.7	8.4%	Annelida	*Spiophanes kroeyeri*	13.6	9.7%	Annelida	*Spiophanes kroeyeri*	20.8	9.3%
		Annelida	*Streblosoma bairdi*	6.3	5.5%	Annelida	*Aphelochaeta marioni*	11.4	8.1%	Mollusca	*Thyasira equalis*	20.2	9.1%
		Mollusca	*Abra nitida*	5.3	4.7%	Annelida	*Chaetozone setosa*	8.8	6.3%	Annelida	*Chaetozone setosa*	19.6	8.8%
		Mollusca	*Ennucula tenuis*	3.7	3.2%	Annelida	*Heteromastus filiformis*	8.0	5.7%	Annelida	*Scalibregma inflatum*	14.4	6.5%
		Annelida	*Aphelochaeta marioni*	3.3	2.9%	Annelida	*Streblosoma bairdi*	6.2	4.4%	Annelida	*Aphelochaeta marioni*	9.0	4.0%
		Annelida	*Abyssoninoe hibernica*	3.0	2.6%	Annelida	*Lipobranchius jeffreysii*	4.2	3.0%	Annelida	*Prionospio dubia*	6.6	3.0%
		Annelida	*Heteromastus filiformis*	2.7	2.3%	Mollusca	*Yoldiella philippiana*	4.0	2.8%	Annelida	*Paramphinome jeffreysi*	6.2	2.8%
						Annelida	*Abyssoninoe hibernica*	3.6	2.6%	Annelida	*Diplocirrus glaucus*	6.2	2.8%
			Sum	76.7	66.9%		Sum	99.0	70.5%		Sum	146.6	65.8%
			Tot	114.7	100%		Tot	140.4	100%		Tot	222.8	100%
	Ref-95					Annelida	*Chaetozone setosa*	31.4	21.9%	Annelida	*Chaetozone setosa*	53.0	20.9%
						Annelida	*Spiophanes kroeyeri*	15.2	10.6%	Annelida	*Scalibregma inflatum*	41.6	16.4%
						Annelida	*Aphelochaeta marioni*	13.8	9.6%	Annelida	*Paramphinome jeffreysi*	36.8	14.5%
						Mollusca	*Thyasira equalis*	13.6	9.5%	Annelida	*Heteromastus filiformis*	22.0	8.7%
						Annelida	*Paramphinome jeffreysi*	13.4	9.3%	Mollusca	*Thyasira equalis*	15.6	6.2%
						Annelida	*Heteromastus filiformis*	8.6	6.0%	Annelida	*Prionospio cirrifera*	14.8	5.8%
						Annelida	*Ceratocephale loveni*	6.2	4.3%	Annelida	*Spiophanes kroeyeri*	13.8	5.5%
						Annelida	*Scalibregma inflatum*	4.6	3.2%	Annelida	*Aphelochaeta marioni*	13.0	5.1%
						Mollusca	*Abra nitida*	4.4	3.1%	Annelida	*Ceratocephale loveni*	8.2	3.2%
						Arthropoda	*Leucon nasica*	3.6	2.5%	Arthropoda	*Eudorella emarginata*	4.0	1.6%
						Sum	Sum	114.8	79.9%		Sum	222.8	88.1%
						Tot	Tot	143.6	100%		Tot	253.0	100%

b)		1 month			14 months			49 months		
		Taxa	**BPc**	**%**	***Taxa***	**BPc**	**%**	***Taxa***	**BPc**	**%**
**30 m**	**AC+clay-30**	*Echinocardium cordatum*	282.8	38.3%	*Brissopsis lyrifera*	61.4	41.0%	*Pectinaria koreni*	188.7	35.1%
		Brissopsis lyrifera	206.0	27.9%	*Nephtys incisa*	19.9	13.3%	*Nucula nitidosa*	88.9	16.5%
		Nephtys incisa	69.1	9.4%	*Abyssoninoe hibernica*	16.0	10.7%	*Nephtys incisa*	86.0	16.0%
		Scalibregma inflatum	39.9	5.4%	*Corbula gibba*	7.7	5.1%	*Abra nitida*	78.5	14.6%
		Corbula gibba	24.3	3.3%	*Scalibregma inflatum*	6.7	4.5%	*Thyasira flexuosa*	28.5	5.3%
		Hyala vitrea	20.6	2.8%	*Notomastus latericeus*	5.8	3.9%	*Goniada maculata*	11.8	2.2%
		Nucula nitidosa	15.2	2.1%	*Chaetopterus norvegicus*	5.4	3.6%	*Hermania scabra*	9.2	1.7%
		Cylichna cylindracea	13.7	1.9%	*Thyasira sarsii*	4.1	2.8%	*Nemertea*	8.2	1.5%
		Abra nitida	8.1	1.1%	*Goniada maculata*	*3.9*	2.6%	*Abyssoninoe hibernica*	7.0	1.3%
		Tellimya tenella	7.2	1.0%	*Abra nitida*	3.9	2.6%	*Corbula gibba*	6.6	1.2%
		Sum	687	93%	Sum	135	90%	Sum	513	95%
		Tot	738	100%	Tot	150	100%	Tot	538	100%
	**Clay-30**	*Amphiura filiformis*	635.0	32.5%	*Amphiura filiformis*	477.9	27.7%	*Amphiura filiformis*	1959.2	66.5%
		Brissopsis lyrifera	447.9	22.9%	*Scalibregma inflatum*	254.8	14.8%	*Brissopsis lyrifera*	272.2	9.2%
		Polyphysia crassa	116.0	5.9%	*Brissopsis lyrifera*	239.8	13.9%	*Echinocardium flavescens*	128.5	4.4%
		Echinocardium cordatum	111.7	5.7%	*Echinocardium cordatum*	194.5	11.3%	*Amphiura chiajei*	122.3	4.2%
		Amphiura chiajei	102.1	5.2%	*Cerebratulus*	111.9	6.5%	*Pectinaria belgica*	67.1	2.3%
		Corbula gibba	88.1	4.5%	*Amphiura chiajei*	84.0	4.9%	*Abyssoninoe hibernica*	47.3	1.6%
		Nucula nitidosa	69.1	3.5%	*Nephtys incisa*	78.1	4.5%	*Cerebratulus*	35.3	1.2%
		Abyssoninoe hibernica	62.4	3.2%	*Corbula gibba*	47.1	2.7%	*Glycera alba*	32.9	1.1%
		Echinocardium flavescens	59.3	3.0%	*Mesothuria intestinalis*	40.6	2.4%	*Nephtys incisa*	29.9	1.0%
		Hyala vitrea	45.1	2.3%	*Thyasira flexuosa*	23.4	1.4%	*Corbula gibba*	26.7	0.9%
		Sum	1737	89%	Sum	1552	90%	Sum	2722	92%
		Tot	1952	100%	Tot	1725	100%	Tot	2945	100%
	**Ref-30**	*Amphiura filiformis*	474.6	38.2%	*Scalibregma inflatum*	300.3	28.8%	*Amphiura filiformis*	1326.8	42.6%
		Brissopsis lyrifera	319.0	25.7%	*Amphiura filiformis*	158.9	15.3%	*Brissopsis lyrifera*	711.9	22.9%
		Echinocardium cordatum	79.4	6.4%	*Brissopsis lyrifera*	136.5	13.1%	*Echinocardium flavescens*	548.9	17.6%
		Corbula gibba	54.7	4.4%	*Echinocardium cordatum*	119.0	11.4%	*Abyssoninoe hibernica*	66.8	2.1%
		Lipobranchius jeffreysii	41.4	3.3%	*Callianassa subterranea*	50.0	4.8%	*Amphiura chiajei*	61.8	2.0%
		Nemertea	40.9	3.3%	*Pectinaria belgica*	43.5	4.2%	*Callianassa subterranea*	52.4	1.7%
		Callianassa subterranea	35.3	2.8%	*Nephtys incisa*	33.1	3.2%	*Cerebratulus*	51.0	1.6%
		Amphiura chiajei	26.5	2.1%	*Abyssoninoe hibernica*	29.5	2.8%	*Praxillella affinis*	41.4	1.3%
		Nephtys incisa	25.4	2.0%	*Nemertea*	26.6	2.6%	*Goniada maculata*	36.1	1.2%
		Abyssoninoe hibernica	25.3	2.0%	*Glycera alba*	19.2	1.8%	*Nephtys incisa*	29.7	1.0%
		Sum	1122	90%	Sum	917	88%	Sum	2927	94%
		Tot	1241	100%	Tot	1042	100%	Tot	3111	100%
**80-95 m**	**AC+clay-95**	*Thyasira equalis*	182.1	35.8%	*Paramphinome jeffreysi*	176.8	21.4%	*Thyasira equalis*	214.6	26.6%
		Yoldiella philippiana	51.7	10.2%	*Thyasira equalis*	132.7	16.1%	*Paramphinome jeffreysi*	150.8	18.7%
		Ceratocephale loveni	40.9	8.0%	*Abra nitida*	122.9	14.9%	*Aphelochaeta marioni*	99.7	12.3%
		Paramphinome jeffreysi	26.4	5.2%	*Glycera alba*	73.1	8.9%	*Abyssoninoe hibernica*	30.4	3.8%
		Scalibregma inflatum	22.3	4.4%	*Brissopsis lyrifera*	71.9	8.7%	*Heteromastus filiformis*	24.9	3.1%
		Phylo norvegica	20.9	4.1%	*Aphelochaeta marioni*	35.2	4.3%	*Yoldiella philippiana*	24.7	3.1%
		Chaetozone setosa	17.8	3.5%	*Ceratocephale loveni*	29.6	3.6%	*Glycera rouxii*	22.8	2.8%
		Aphelochaeta marioni	17.7	3.5%	*Glycera rouxii*	20.0	2.4%	*Leucon nasica*	21.9	2.7%
		Spiophanes kroeyeri	17.4	3.4%	*Arrhis phyllonyx*	20.0	2.4%	*Glycera alba*	21.7	2.7%
		Rhodine loveni	13.3	2.6%	*Hermania scabra*	15.4	1.9%	*Amphiura chiajei*	21.7	2.7%
		Sum	410	81%	Sum	698	85%	Sum	633	78%
		Tot	508	100%	Tot	825	100%	Tot	808	100%
	**Ref-80**	*Brissopsis lyrifera*	396.2	33.0%	*Lipobranchius jeffreysii*	315.8	20.5%	*Scalibregma inflatum*	261.9	14.4%
		Thyasira equalis	134.0	11.2%	*Paramphinome jeffreysi*	151.8	9.9%	*Lipobranchius jeffreysii*	146.2	8.0%
		Streblosoma bairdi	87.6	7.3%	*Thyasira equalis*	120.6	7.8%	*Brissopsis lyrifera*	142.9	7.9%
		Lipobranchius jeffreysii	67.2	5.6%	*Aphelochaeta marioni*	92.3	6.0%	*Thyasira equalis*	139.6	7.7%
		Abyssoninoe hibernica	58.2	4.8%	*Brissopsis lyrifera*	84.8	5.5%	*Amphiura chiajei*	128.9	7.1%
		Glycera alba	43.1	3.6%	*Streblosoma bairdi*	77.5	5.0%	*Heteromastus filiformis*	124.2	6.8%
		Glycera rouxii	39.9	3.3%	*Abyssoninoe hibernica*	74.0	4.8%	*Streblosoma bairdi*	91.8	5.1%
		Ennucula tenuis	37.1	3.1%	*Glycera alba*	57.7	3.8%	*Aphelochaeta marioni*	71.9	4.0%
		Yoldiella philippiana	33.6	2.8%	*Amphiura chiajei*	57.3	3.7%	*Chaetozone setosa*	71.2	3.9%
		Aphelochaeta marioni	25.7	2.1%	*Glycera rouxii*	55.3	3.6%	*Glycera alba*	64.7	3.6%
		Sum	923	77%	Sum	1087	71%	Sum	1243	68.4%
		Tot	1200	100%	Tot	1540	100%	Tot	1817	100%
	**Ref-95**				*Aphelochaeta marioni*	134.8	11.8%	*Scalibregma inflatum*	570.6	30.8%
					Chaetozone setosa	132.0	11.5%	*Paramphinome jeffreysi*	267.9	14.5%
					Thyasira equalis	112.3	9.8%	*Chaetozone setosa*	158.8	8.6%
					Scalibregma inflatum	108.7	9.5%	*Ceratocephale loveni*	140.1	7.6%
					Brissopsis lyrifera	92.0	8.0%	*Thyasira equalis*	132.3	7.1%
					Ceratocephale loveni	76.8	6.7%	*Heteromastus filiformis*	91.6	4.9%
					Paramphinome jeffreysi	72.6	6.3%	*Aphelochaeta marioni*	85.7	4.6%
					Glycera rouxii	54.9	4.8%	*Brissopsis lyrifera*	83.1	4.5%
					Abra nitida	45.3	3.9%	*Nemertea*	45.1	2.4%
					Glycera alba	39.5	3.4%	*Goniada maculata*	36.6	2.0%
		Sum			Sum	869	76%	Sum	1612	87%
		Tot			Tot	1147	100%	Tot	1852	100%

c)		1 month			14 months			49 months		
		**Taxa**	**BIPc**	**%**	**Taxa**	**BIPc**	**%**	**Taxa**	**BIPc**	**%**
**30 m**	**AC+clay-30**	*Brissopsis lyrifera*	160.6	27.9%	*Chaetopterus norvegicus*	108.6	50.9%	*Pectinaria koreni*	391.4	74.0%
		Echinocardium cordatum	154.3	26.8%	*Brissopsis lyrifera*	47.9	22.4%	*Nucula nitidosa*	53.5	10.1%
		Scalibregma inflatum	134.6	23.3%	*Scalibregma inflatum*	22.7	10.7%	*Thyasira flexuosa*	17.1	3.2%
		Hyala vitrea	23.5	4.1%	*Notomastus latericeus*	9.8	4.6%	*Nephtys incisa*	16.7	3.2%
		Callianassa subterranea	17.0	2.9%	*Corbula gibba*	4.2	1.9%	*Heteromastus filiformis*	15.4	2.9%
		Nephtys incisa	13.5	2.3%	*Spiophanes kroeyeri*	4.0	1.9%	*Abra nitida*	14.2	2.7%
		Corbula gibba	13.2	2.3%	*Nephtys incisa*	3.9	1.8%	*Corbula gibba*	3.6	0.7%
		Heteromastus filiformis	11.1	1.9%	*Thyasira sarsii*	3.5	1.6%	*Diplocirrus glaucus*	2.6	0.5%
		Tellimya tenella	10.8	1.9%	*Abyssoninoe hibernica*	*3.0*	1.4%	*Thysanocardia procera*	2.3	0.4%
		Nucula nitidosa	9.2	1.6%	*Amphiura filiformis*	1.9	0.9%	*Notomastus latericeus*	1.8	0.3%
		Sum	548	95.0%	Sum	209	98.2%	Sum	519	98.1%
		Tot	577	100%	Tot	213	100%	Tot	529	100%
	**Clay-30**	*Amphiura filiformis*	414.6	24.3%	*Scalibregma inflatum*	858.4	49.9%	*Amphiura filiformis*	1279.3	56.1%
		Polyphysia crassa	390.9	22.9%	*Amphiura filiformis*	312.1	18.1%	*Brissopsis lyrifera*	212.2	9.3%
		Brissopsis lyrifera	349.2	20.4%	*Brissopsis lyrifera*	186.9	10.9%	*Pectinaria belgica*	194.9	8.5%
		Pectinaria belgica	92.1	5.4%	*Echinocardium cordatum*	106.1	6.2%	*Scalibregma inflatum*	79.8	3.5%
		Echinocardium cordatum	60.9	3.6%	*Chaetopterus norvegicus*	35.7	2.1%	*Echinocardium flavescens*	70.1	3.1%
		Hyala vitrea	51.5	3.0%	*Corbula gibba*	25.5	1.5%	*Lipobranchius jeffreysii*	67.3	3.0%
		Corbula gibba	47.7	2.8%	*Amphiura chiajei*	25.1	1.5%	*Praxillella affinis*	62.3	2.7%
		Nucula nitidosa	41.6	2.4%	*Pectinaria belgica*	19.9	1.2%	*Callianassa subterranea*	47.7	2.1%
		Callianassa subterranea	32.5	1.9%	*Glycera alba*	18.0	1.0%	*Chaetopterus norvegicus*	41.1	1.8%
		Echinocardium flavescens	32.4	1.9%	*Nephtys incisa*	15.2	0.9%	*Amphiura chiajei*	36.5	1.6%
		Sum	1513	88.6%	Sum	1603	93.2%	Sum	2091	91.7%
		Tot	1707	100%	Tot	1720	100%	Tot	2281	100%
	**Ref-30**	*Amphiura filiformis*	309.9	31.0%	*Scalibregma inflatum*	1011.7	53.6%	*Amphiura filiformis*	866.4	35.7%
		Brissopsis lyrifera	248.6	24.9%	*Callianassa subterranea*	131.2	7.0%	*Brissopsis lyrifera*	554.9	22.9%
		Lipobranchius jeffreysii	139.6	14.0%	*Pectinaria belgica*	126.4	6.7%	*Echinocardium flavescens*	299.5	12.4%
		Callianassa subterranea	92.6	9.3%	*Chaetopterus norvegicus*	120.7	6.4%	*Praxillella affinis*	268.1	11.1%
		Echinocardium cordatum	43.3	4.3%	*Brissopsis lyrifera*	106.4	5.6%	*Callianassa subterranea*	137.5	5.7%
		Praxillella praetermissa	41.6	4.2%	*Amphiura filiformis*	103.7	5.5%	*Lipobranchius jeffreysii*	74.0	3.1%
		Corbula gibba	29.6	3.0%	*Echinocardium cordatum*	64.9	3.4%	*Scalibregma inflatum*	73.2	3.0%
		Eriopisa elongata	22.6	2.3%	*Praxillella praetermissa*	59.8	3.2%	*Pectinaria belgica*	23.7	1.0%
		Spiophanes kroeyeri	13.6	1.4%	*Praxillella affinis*	25.0	1.3%	*Amphiura chiajei*	18.5	0.8%
		Thysanocardia procera	12.3	1.2%	*Spiophanes kroeyeri*	21.9	1.2%	*Glycera alba*	16.3	0.7%
		Sum	954	95.5%	Sum	1772	93.9%	Sum	2332	96.2%
		Tot	999	100%	Tot	1887	100%	Tot	2424	100%
**80-95 m**	**AC+clay-95**	*Rhodine loveni*	128.9	22.9%	*Rhodine loveni*	85.2	17.4%	*Thyasira equalis*	129.2	26.6%
		Thyasira equalis	109.6	19.5%	*Thyasira equalis*	79.9	16.3%	*Spiophanes kroeyeri*	71.5	14.7%
		Scalibregma inflatum	75.2	13.4%	*Glycera alba*	58.9	12.0%	*Heteromastus filiformis*	63.0	13.0%
		Spiophanes kroeyeri	73.3	13.0%	*Spiophanes kroeyeri*	57.7	11.8%	*Scalibregma inflatum*	44.6	9.2%
		Neoamphitrite grayi	31.6	5.6%	*Brissopsis lyrifera*	56.0	11.4%	*Aphelochaeta marioni*	27.3	5.6%
		Phylo norvegica	26.5	4.7%	*Heteromastus filiformis*	28.8	5.9%	*Paramphinome jeffreysi*	23.3	4.8%
		Melinna cristata	18.2	3.2%	*Paramphinome jeffreysi*	27.3	5.6%	*Glycera rouxii*	18.4	3.8%
		Heteromastus filiformis	15.7	2.8%	*Abra nitida*	22.2	4.5%	*Glycera alba*	17.5	3.6%
		Pista cristata	12.3	2.2%	*Glycera rouxii*	16.2	3.3%	*Eudorella emarginata*	16.5	3.4%
		Ceratocephale loveni	8.8	1.6%	*Aphelochaeta marioni*	9.6	2.0%	*Prionospio cirrifera*	11.2	2.3%
		Sum	500	88.9%	Sum	442	90.3%	Sum	423	87.0%
		Tot	562	100%	Tot	489	100%	Tot	486	100%
	**Ref-80**	*Brissopsis lyrifera*	308.9	20.7%	*Lipobranchius jeffreysii*	1063.9	44.4%	*Scalibregma inflatum*	882.3	28.1%
		Streblosoma bairdi	289.4	19.4%	*Streblosoma bairdi*	256.0	10.7%	*Lipobranchius jeffreysii*	492.7	15.7%
		Lipobranchius jeffreysii	226.5	15.2%	*Rhodine loveni*	254.1	10.6%	*Rhodine loveni*	323.6	10.3%
		Spiophanes kroeyeri	93.0	6.2%	*Scalibregma inflatum*	134.6	5.6%	*Heteromastus filiformis*	314.6	10.0%
		Thyasira equalis	80.7	5.4%	*Neoamphitrite affinis*	101.3	4.2%	*Streblosoma bairdi*	303.2	9.7%
		Rhodine loveni	66.7	4.5%	*Heteromastus filiformis*	78.3	3.3%	*Brissopsis lyrifera*	111.4	3.6%
		Pectinaria belgica	64.4	4.3%	*Thyasira equalis*	72.6	3.0%	*Spiophanes kroeyeri*	101.9	3.2%
		Neoamphitrite affinis	62.2	4.2%	*Brissopsis lyrifera*	66.1	2.8%	*Thyasira equalis*	84.0	2.7%
		Glycera alba	34.7	2.3%	*Spiophanes kroeyeri*	62.3	2.6%	*Praxillella affinis*	75.9	2.4%
		Heteromastus filiformis	34.3	2.3%	*Glycera alba*	46.5	1.9%	*Polyphysia crassa*	65.9	2.1%
		Sum	1261	84.6%	Sum	2136	89.2%	Sum	2756	87.9%
		Tot	1491	100%	Tot	2395	100%	Tot	3136	100%
	**Ref-95**				*Scalibregma inflatum*	366.3	30.3%	*Scalibregma inflatum*	882.3	28.1%
					Rhodine loveni	138.1	11.4%	*Lipobranchius jeffreysii*	492.7	15.7%
					Spiophanes kroeyeri	127.7	10.6%	*Rhodine loveni*	323.6	10.3%
					Heteromastus filiformis	90.0	7.4%	*Heteromastus filiformis*	314.6	10.0%
					Brissopsis lyrifera	71.7	5.9%	*Streblosoma bairdi*	303.2	9.7%
					Thyasira equalis	67.6	5.6%	*Brissopsis lyrifera*	111.4	3.6%
					Chaetozone setosa	54.3	4.5%	*Spiophanes kroeyeri*	101.9	3.2%
					Glycera rouxii	44.2	3.7%	*Thyasira equalis*	84.0	2.7%
					Aphelochaeta marioni	37.0	3.1%	*Praxillella affinis*	75.9	2.4%
					Glycera alba	31.9	2.6%	*Polyphysia crassa*	65.9	2.1%
					Sum	1029	85.0%	Sum	2756	87.9%
					Tot	1210	100%	Tot	3136	100%

d)		1 month			14 months			49 months		
		Taxa	**IPc**	**%**	***Taxa***	**IPc**	**%**	***Taxa***	**IPc**	**%**
**30 m**	**AC+clay-30**	*Echinocardium cordatum*	74.1	42.5%	*Brissopsis lyrifera*	19.3	44.1%	*Pectinaria koreni*	29.7	42.1%
		Brissopsis lyrifera	65.0	37.3%	*Chaetopterus norvegicus*	13.6	31.2%	*Nephtys incisa*	15.2	21.5%
		Nephtys incisa	11.8	6.8%	*Nephtys incisa*	3.2	7.3%	*Nucula nitidosa*	12.9	18.3%
		Scalibregma inflatum	6.4	3.7%	*Abyssoninoe hibernica*	1.9	4.4%	*Abra nitida*	4.6	6.6%
		Nucula nitidosa	5.1	2.9%	*Notomastus latericeus*	1.8	4.1%	Nemertea	1.1	1.6%
		Trichobranchus roseus	2.2	1.2%	*Spiophanes kroeyeri*	0.9	2.0%	*Goniada maculata*	1.1	1.6%
		Hyala vitrea	1.7	1.0%	*Scalibregma inflatum*	0.8	1.7%	*Leptopentacta elongata*	1.0	1.4%
		Callianassa subterranea	1.2	0.7%	*Golfingia vulgaris*	0.3	0.8%	*Heteromastus filiformis*	0.9	1.3%
		Corbula gibba	1.0	0.6%	Cerebratulus	*0.3*	0.7%	*Abyssoninoe hibernica*	0.9	1.3%
		Cylichna cylindracea	1.0	0.6%	*Hermania scabra*	0.3	0.7%	*Hermania scabra*	0.7	1.0%
		Sum	170	97.2%	Sum	42	97.1%	Sum	68	96.6%
		Tot	174	100%	Tot	44	100%	Tot	71	100%
	**Clay-30**	*Brissopsis lyrifera*	147.8	40.5%	*Brissopsis lyrifera*	67.2	22.5%	*Amphiura filiformis*	255.3	54.4%
		Amphiura filiformis	71.8	19.7%	*Amphiura filiformis*	56.7	19.0%	*Brissopsis lyrifera*	81.6	17.4%
		Polyphysia crassa	30.7	8.4%	*Echinocardium cordatum*	48.6	16.3%	*Echinocardium flavescens*	30.7	6.5%
		Echinocardium cordatum	25.9	7.1%	Cerebratulus	44.6	14.9%	*Pectinaria belgica*	22.3	4.8%
		Amphiura chiajei	14.4	3.9%	*Scalibregma inflatum*	21.5	7.2%	*Amphiura chiajei*	14.4	3.1%
		Echinocardium flavescens	14.2	3.9%	*Nephtys incisa*	15.4	5.2%	Cerebratulus	11.8	2.5%
		Pectinaria belgica	10.6	2.9%	*Amphiura chiajei*	12.9	4.3%	*Abyssoninoe hibernica*	6.1	1.3%
		Nucula nitidosa	10.3	2.8%	*Golfingia vulgaris*	2.8	0.9%	*Lipobranchius jeffreysii*	5.5	1.2%
		Abyssoninoe hibernica	7.5	2.0%	*Nucula nitidosa*	2.6	0.9%	*Nephtys incisa*	4.2	0.9%
		Antalis entalis	3.7	1.0%	*Chaetopterus norvegicus*	2.6	0.9%	*Callianassa subterranea*	3.3	0.7%
		Sum	337	92.2%	Sum	275	92.0%	Sum	435	92.8%
		Tot	365	100%	Tot	299	100%	Tot	469	100%
	**Ref-30**	*Brissopsis lyrifera*	89.4	39.3%	*Brissopsis lyrifera*	45.6	22.2%	*Brissopsis lyrifera*	221.0	35.8%
		Amphiura filiformis	54.3	23.9%	*Echinocardium cordatum*	30.0	14.6%	*Amphiura filiformis*	178.0	28.9%
		Echinocardium cordatum	22.0	9.7%	*Scalibregma inflatum*	29.0	14.1%	*Echinocardium flavescens*	105.2	17.1%
		Nemertea	13.3	5.9%	*Amphiura filiformis*	17.2	8.4%	Cerebratulus	23.8	3.9%
		Lipobranchius jeffreysii	12.7	5.6%	*Pectinaria belgica*	16.3	7.9%	*Callianassa subterranea*	14.0	2.3%
		Callianassa subterranea	8.0	3.5%	*Chaetopterus norvegicus*	16.0	7.8%	*Praxillella affinis*	13.9	2.3%
		Nephtys incisa	4.2	1.8%	*Callianassa subterranea*	10.3	5.0%	*Abyssoninoe hibernica*	8.3	1.3%
		Amphiura chiajei	3.0	1.3%	Nemertea	6.2	3.0%	*Golfingia vulgaris*	7.5	1.2%
		Spiophanes kroeyeri	3.0	1.3%	*Nephtys incisa*	5.8	2.8%	*Amphiura chiajei*	7.0	1.1%
		Abyssoninoe hibernica	3.0	1.3%	*Spiophanes kroeyeri*	4.8	2.3%	*Lipobranchius jeffreysii*	6.3	1.0%
		Sum	213	93.5%	Sum	181	88.2%	Sum	585	94.9%
		Tot	228	100%	Tot	205	100%	Tot	617	100%
**80-95 m**	**AC+clay-95**	*Spiophanes kroeyeri*	15.3	20.4%	*Brissopsis lyrifera*	24.4	26.9%	*Spiophanes kroeyeri*	12.8	16.7%
		Neoamphitrite grayi	11.2	14.9%	*Spiophanes kroeyeri*	10.1	11.2%	*Paramphinome jeffreysi*	8.7	11.4%
		Rhodine loveni	6.7	8.9%	*Paramphinome jeffreysi*	9.8	10.7%	*Aphelochaeta marioni*	8.6	11.3%
		Pista cristata	6.4	8.6%	*Glycera alba*	7.5	8.2%	*Glycera rouxii*	4.4	5.7%
		Phylo norvegica	6.1	8.2%	*Abra nitida*	4.6	5.1%	*Abyssoninoe hibernica*	3.8	4.9%
		Ceratocephale loveni	5.3	7.1%	*Scoletoma fragilis*	4.3	4.7%	*Thyasira equalis*	3.6	4.7%
		Yoldiella philippiana	3.1	4.1%	*Glycera rouxii*	3.6	4.0%	*Heteromastus filiformis*	3.6	4.7%
		Scalibregma inflatum	2.9	3.9%	*Rhodine loveni*	3.6	3.9%	*Prionospio cirrifera*	3.5	4.6%
		Thyasira equalis	2.8	3.7%	*Ceratocephale loveni*	3.3	3.7%	*Amphiura chiajei*	2.9	3.8%
		Melinna cristata	2.5	3.3%	*Arrhis phyllonyx*	3.3	3.7%	*Eudorella emarginata*	2.6	3.3%
		Sum	62	82.9%	Sum	75	82.0%	Sum	55	71.1%
		Tot	75	100%	Tot	91	100%	Tot	77	100%
	**Ref-80**	*Streblosoma bairdi*	123.3	31.4%	*Streblosoma bairdi*	105.7	28.8%	*Streblosoma bairdi*	145.2	33.5%
		Brissopsis lyrifera	112.4	28.6%	*Lipobranchius jeffreysii*	76.0	20.7%	*Brissopsis lyrifera*	39.7	9.2%
		Neoamphitrite affinis	30.9	7.9%	*Neoamphitrite affinis*	41.5	11.3%	*Lipobranchius jeffreysii*	39.0	9.0%
		Lipobranchius jeffreysii	18.6	4.7%	*Brissopsis lyrifera*	22.1	6.0%	*Scalibregma inflatum*	31.0	7.2%
		Pista cristata	15.7	4.0%	*Rhodine loveni*	13.6	3.7%	*Heteromastus filiformis*	22.4	5.2%
		Spiophanes kroeyeri	15.4	3.9%	*Abyssoninoe hibernica*	10.3	2.8%	*Amphiura chiajei*	16.5	3.8%
		Scoletoma fragilis	12.8	3.3%	*Glycera rouxii*	10.1	2.8%	*Spiophanes kroeyeri*	15.8	3.7%
		Pectinaria belgica	9.4	2.4%	*Spiophanes kroeyeri*	9.3	2.5%	*Rhodine loveni*	15.6	3.6%
		Abyssoninoe hibernica	7.7	2.0%	*Cerebratulus*	9.2	2.5%	*Nemertea*	7.5	1.7%
		Ennucula tenuis	6.0	1.5%	*Paramphinome jeffreysi*	8.3	2.3%	*Chaetozone setosa*	7.5	1.7%
		Sum	352	89.7%	Sum	306	83.4%	Sum	340	78.6%
		Tot	393	100%	Tot	367	100%	Tot	433	100%
	**Ref-95**				*Brissopsis lyrifera*	35.4	19.4%	*Scalibregma inflatum*	58.8	23.3%
					Spiophanes kroeyeri	25.6	14.1%	*Brissopsis lyrifera*	30.4	12.0%
					Chaetozone setosa	15.0	8.2%	*Ceratocephale loveni*	19.6	7.8%
					Scalibregma inflatum	14.7	8.0%	*Paramphinome jeffreysi*	16.1	6.4%
					Aphelochaeta marioni	12.7	7.0%	*Chaetozone setosa*	15.3	6.1%
					Glycera rouxii	10.6	5.8%	*Heteromastus filiformis*	14.4	5.7%
					Ceratocephale loveni	9.1	5.0%	*Streblosoma bairdi*	14.3	5.6%
					Rhodine loveni	7.2	4.0%	*Spiophanes kroeyeri*	10.6	4.2%
					Pista cristata	5.6	3.1%	*Polyphysia crassa*	8.8	3.5%
					Heteromastus filiformis	5.5	3.0%	*Nemertea*	8.1	3.2%
					Sum	141	77.6%	Sum	196	77.7%
					Tot	182	100%	Tot	253	100%
